# TTC36 promotes proliferation and drug resistance in hepatocellular carcinoma cells by inhibiting c-Myc degradation

**DOI:** 10.1038/s41419-025-07663-4

**Published:** 2025-04-24

**Authors:** Fengling Shao, Runzhi Wang, Xinyi Li, Yanxia Hu, Zaikuan Zhang, Jing Cai, Jieru Yang, Xiaosong Feng, Suxia Ren, Zengyi Huang, Yajun Xie

**Affiliations:** 1 The Ministry of Education Key Laboratory of Laboratory Medical Diagnostics, the College of Laboratory Medicine, Chongqing Medical University, Chongqing, China; 2 School of Life and Health Sciences, Hainan University, Haikou, China; 3 College of Basic Medical Sciences, Harbin Medical University, Harbin, China; 4 Department of Cell Biology and Genetics, School of Basic Medical Sciences, Chongqing Medical University, Chongqing, China; 5 Mitomedical laboratory of Children’s Hospital of Chongqing Medical University, National Clinical Research Center for Child Health and Disorders, Ministry of Education Key Laboratory of Child Development and Disorders, Chongqing Key Laboratory of Child Rare Diseases in Infection and Immunity, Chongqing, China

**Keywords:** Molecular biology, Gastrointestinal cancer

## Abstract

High c-Myc protein accumulation contributes to the proliferation, invasion, and drug resistance in multiple cancer cells, but the underlying mechanism about c-Myc accumulation remains not to be elucidated. Here, we demonstrate that TTC36 promotes c-Myc protein accumulation in hepatocellular carcinoma cells, thereby driving the proliferation and sorafenib resistance in hepatocellular carcinoma cells. *Ttc36* depletion disrupts the interaction between SET and PPP2R1A, consequently activating PP2A. Activated PP2A directly dephosphorylates p-c-Myc^S62^ and activates GSK3β, relying on AKT, leading increased phosphorylation of p-c-Myc^T58^, finally promotes FBXW7-mediated polyubiquitination and degradation of c-Myc. Inhibitors targeting GSK3β and PP2A effectively reverse the sorafenib resistance promoted by TTC36. These findings highlight the crucial role of TTC36 in c-Myc accumulation-caused proliferation and sorafenib resistance in HCC, providing a promising combination strategy for treating patients with c-Myc protein accumulation in advanced HCC.

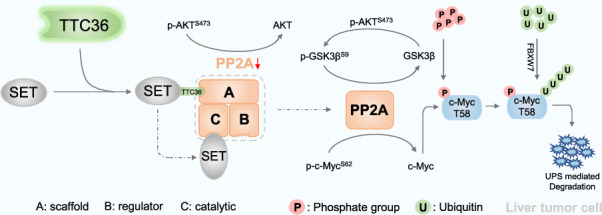

## Introduction

Hepatocellular carcinoma (HCC), ranked as the third leading cause of cancer-related mortality, is the most frequent primary liver cancer, accounting for ~90% cases, and is characterized by both phenotypic and molecular heterogeneity [1]. The main risk factors for HCC development include infection by hepatitis B virus and hepatitis C virus [1]. Sorafenib, a multi-target tyrosine kinase inhibitor with the ability to induce apoptosis, inhibit angiogenesis, and suppress tumor cell proliferation, has been established as an effective first-line therapy for advanced HCC, prolonging the overall median survival of patients [2]. Patients benefiting from sorafenib treatment frequently develop drug resistance, resulting in a high rate of recurrence that poses a significant challenge in the battle against HCC [3]. While both immunotherapy and molecular therapies for HCC need to be further developed in China [4, 5], understanding and elucidating the mechanisms of sorafenib resistance in HCC are crucial for the advancement of novel drugs and therapeutic strategies.

Focal chromosome amplifications in loci of *CCND1*, *FGF19*, *VEGFA*, *MYC,* or *MET* result in the activation of various oncogenic signaling pathways in HCC initiation [6]. c-Myc, an undruggable target, has been found to be overexpressed in 75% of cancers [7], with genetic locus amplification occurring in 18–25% of human HCC samples [8–11]. Previous studies have established that c-Myc contributes to HCC progression and the oncogenic reprogramming of adult hepatocytes into cancer stem cells (CSCs) [12]. Therefore, targeting pathways that result in the degradation of c-Myc, especially in combination with traditional therapies, is considered an attractive therapeutic strategy for HCC. c-Myc accumulation contributes to maintaining the stemness of HCC cells, which have been shown to correlate with drug resistance in HCC [13–15]. Importantly, some studies have revealed that c-Myc can be degraded by the ubiquitin-proteasome system [16], the phosphorylation of c-Myc at Thr 58 and Ser 62 plays a pivotal role for c-Myc accumulation [17, 18]. Exploring new modifiers in c-Myc accumulation and investigating the underlying mechanisms is crucial for the development of strategies in HCC treatment. Our earlier research revealed that TTC36 regulates the ubiquitination and degradation of HPD, resulting in hypertyrosinemia in mice [19]. Be consistent with previous studies, TTC36 is predominantly expressed in liver [20]. However, the involvement of TTC36 protein accumulation in HCC progression remains unexplored.

Here, a reduction in c-Myc as observed in *Ttc36*^−/−^ mice without mRNA expression was changed. Our findings indicate that TTC36 promotes the proliferation and sorafenib resistance in HCC. TTC36 deactivates PP2A by promoting the interaction between SET and the subunit of PP2A complex, leading to the prevention of polyubiquitination-mediated degradation of c-Myc protein. These findings highlight the underlying mechanism of TTC36’s role in regulating c-Myc accumulation and provide new insights into the TTC36/c-Myc pathway in HCC progression, suggesting novel approaches for innovative treatment strategies.

## Results

### TTC36 promotes the proliferation and cell cycle of HCC cells

**Fig. 1:**
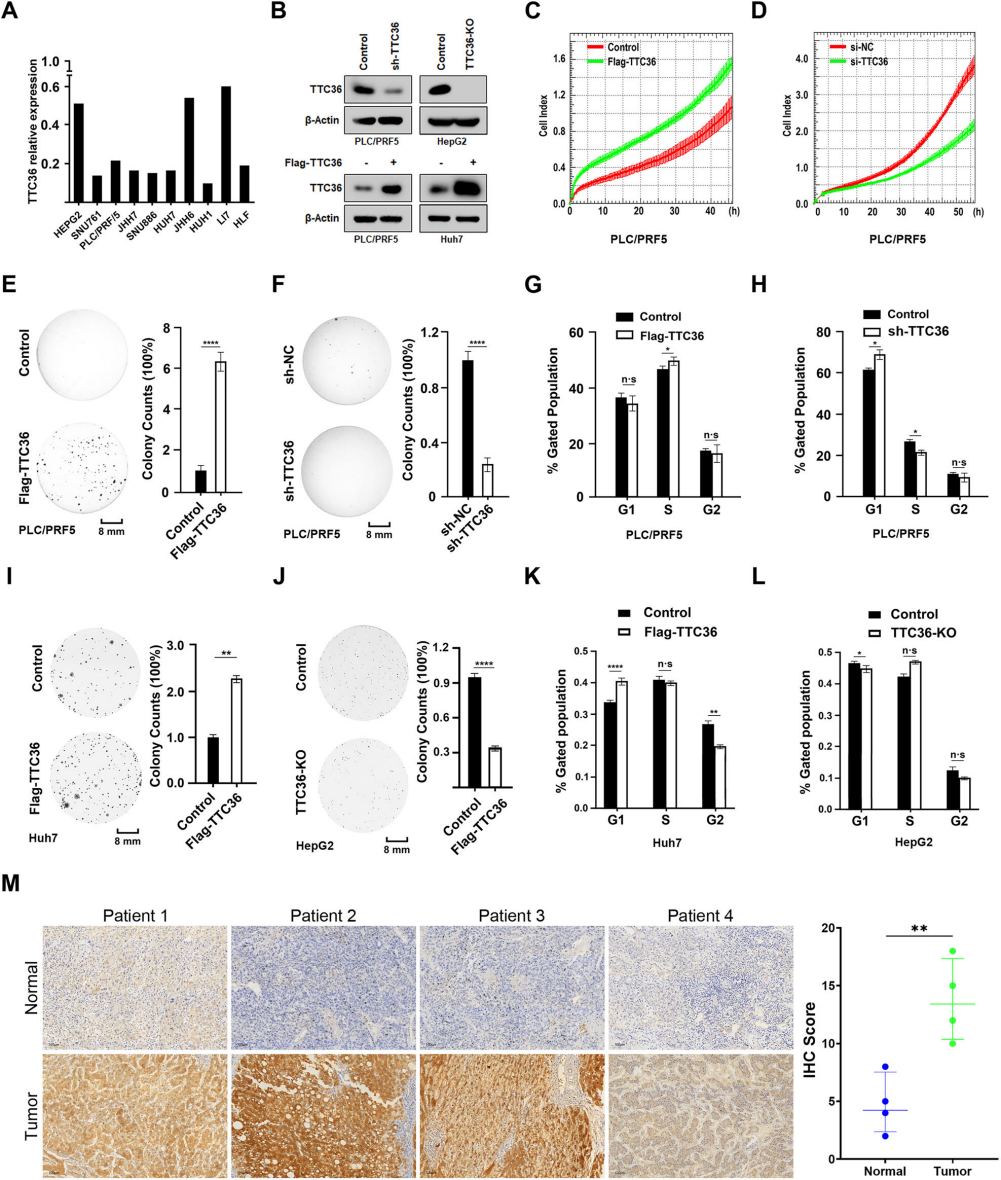
TTC36 promotes cell proliferation and induces the migration and cell cycle of hepatocellular carcinoma cells. **A** Expression levels of TTC36 across various cell lines. **B** Cell lysates were extracted from Flag-TTC36-overexpressing Huh7 and PCL/PRF/5, TTC36-knockdown in PCL/PRF/5, and TTC36-knockout in HepG2 cells, followed by immunoblotting with antibodies against TTC36; β-actin served as the loading control. **C**, **D** In vitro growth of PLC/PRF/5 cells treated with Flag-TTC36 or TTC36 RNAi was assessed through Real-Time Continuous Assessment. **E**, **F** Colony Formation assay was performed on PLC/PRF/5 cells with Flag-TTC36 or sh-TTC36 (left panel), and the corresponding quantitative analysis is shown on the right (mean ± SD (*n* = 4)). ****p < 0.0001. **G**, **H** Cell cycle analysis of PLC/PRF/5 cells was conducted using flow cytometry with propidium iodide staining, revealing the percentage of cells in different cell cycle phases. The results are displayed with error bars representing mean ± SD (*n* = 3). *p < 0.05. **I**, **J** Colony formation assays were conducted on Huh7 or HepG2 cells expressing Flag-TTC36 or Sg-TTC36 (left panel), with quantitative analyses on the right (mean ± SD (*n* = 3)). **p < 0.01, ****p < 0.0001. **K**, **L** Cell cycle analysis of Huh7 or HepG2 cells performed through flow cytometry and propidium iodide staining, indicating the percentage of cells in different cell cycle phases. Results are presented with error bars, representing mean ± SD (*n* = 3). *p < 0.05, **p < 0.01, ****p < 0.0001. **M** IHC score levels of TTC36 in tumor and normal tissues of HCC patients.

To investigate the role of TTC36 in promoting HCC, we analyzed the mRNA expression level of *TTC36* in various HCC cell lines. We selected PLC/PRF/5 (medium TTC36 level), Huh7 (low TTC36 level) and HepG2 (high TTC36 level) cell lines to further study the function of TTC36 (Fig. 1A). Using the CRISPR/Cas9 system, a *Ttc36* knockout cell line was established in HepG2 cells, stable *Ttc36* knockdown cells were screened in PLC/PRF/5 and stable *Ttc36* overexpresed cells were screened in PLC/PRF/5 and Huh7 cells through lentivirus -mediated transfection (Fig. 1B). Expression manipulation of Ttc36 in PLC/PRF/5 cells was performed by overexpression and shRNA-mediated knockdown in PLC/PRF/5 cells. Overexpression of *Ttc36* resulted in a significant increase in cell growth compared to the control cells (Figs. 1C and S1A, Supporting Information), while knockdown of *Ttc36* inhibited cell growth (Figs. 1D and S1B, Supporting Information). Soft agar assays validated the effects of cell non-anchorage-dependent growth induced by *Ttc3*6 (Fig. 1E, F). Flow cytometry demonstrated that overexpression of *Ttc36* lead to a significant increase in S-phase cell numbers (Fig. 1G), while knocking down of *Ttc36* decreased S-phase cell numbers and increased G1-phase cell numbers (Fig. 1H), which suggests that TTC36 may regulate PLC/PRF/5 cell proliferation mainly by regulating S phase. Notably, the expresion level of *Ttc36* have no effect on cell apoptosis (Fig. S1E, S1F, Supporting Information). Scratch-wound healing assays suggested that overexpression of *Ttc36* promoted cell migration (Fig. S1G, Supporting Information), while knockdown of *Ttc36* resulted in a defect in cell migration (Fig. S1H, Supporting Information). These findings indicate that TTC36 plays important roles in cell growth, proliferation, and migration in HCC cells.

To further validate the role of TTC36 in promoting HCC, HUH7 cells with lower expression levels of *Ttc36* than SNU761 and HepG2 cells were selected for further study (Fig. 1A). Consistent with the findings in PLC/PRF/5 cells, overexpression of *Ttc36* significantly promoted cell growth in Huh7 cells (Figs. 1I and S1C, Supporting Information), while deficiency of *Ttc36* inhibited cell growth in HepG2 cells (Figs. 1J and S1D, Supporting Information). Flow cytometry analysis revealed that overexpression of *Ttc36* resulted in a marked increase in G1-phase cell numbers and a decrease in G2-phase cell numbers (Fig. 1K), while knocking down *Ttc36* reduced G1-phase cell numbers and increased S-phase cell numbers (Fig. 1L), which The proliferation regulation of Huh7 and HepG2 by TTC36 may be in the G1 phase of cells. Importantly, Ttc36 did not significantly affect cell death in Huh7 and HepG2 cells (Fig. S1I and S1J, Supporting Information). Wound healing assays indicated that Ttc36 overexpression increased wound healing (Fig. S1K, Supporting Information), while Ttc36 knockdown resulted in decreased wound healing (Fig. S1L, Supporting Information).

In summary, the findings indicate that TTC36, which is highly expressed in HCC (Fig. 1M), exerts a tumor-promoting function in HCC by facilitating cell proliferation, advancing cell cycle progression, and enhancing migration, rather than triggering apoptosis.

### TTC36 Stablizes c-Myc Protein by Impeding Ubiquitin-mediated Degradation

**Fig. 2:**
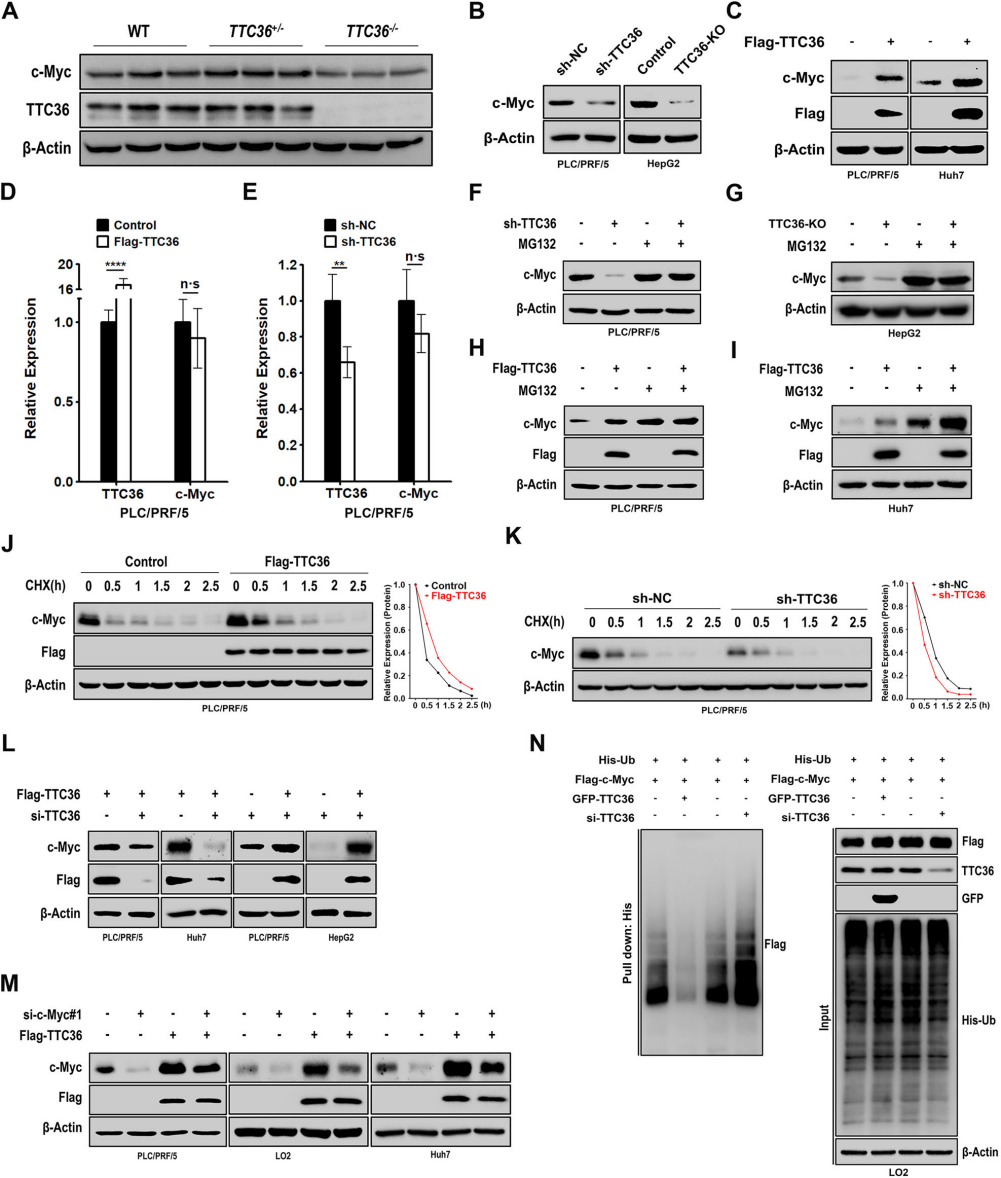
TTC36 Modulates c-Myc Expression and Ubiquitylation. **A** Immunoblotting of liver lysates from wild-type (WT) and TTC36^−/−^ C57BL/6 mice using antibodies against TTC36 and c-Myc; β-Actin served as the loading control. **B** Western blotting of TTC36 knockdown PLC/PRF/5 or TTC36 knockout HepG2 cells was performed using c-Myc antibody; β-actin was a loading control. **C** Immunoblotting of PLC/PRF/5 or Huh7 cells transfected with a plain vector or TTC36-encoding plasmid using indicated antibodies. **D**, **E** Q-PCR analysis of TTC36 and c-Myc mRNA levels in PLC/PRF/5, normalized to 18S, with error bars representing mean ± SD (*n* = 3). *p < 0.05, **p < 0.01. **F**, **G** Western blot analysis of PLC/PRF/5 cells with TTC36 knockdown or HepG2 with TTC36 knockout, treated with or without MG-132 (10 μM) for 6 h at indicated time points. **H**, **I** Western blot analysis of PLC/PRF/5 or Huh7 cells with Flag-TTC36 overexpression, treated with or without MG-132 (10 μM) for 6 h at indicated time points. **J**, **K** Immunoblotting analysis of PLC/PRF/5 cells expressing Flag-TTC36 or TTC36 knockdown, treated with 10 μg mL-1 CHX at indicated time points. **L** Western blot analysis of PLC/PRF/5 or Huh7 cells with or not with si-TTC36 and based on stable Flag-TTC36 overexpression, PLC/PRF/5 or HepG2 cells with or not with Flag-TTC36 overexpression and based on si-TTC36, to observe changes in c-Myc protein levels. **M** Western blot analysis of PLC/PRF/5 cells with c-Myc knockdown or Flag-TTC36 overexpression, or co-transfection, to observe changes in c-Myc protein levels. **N** HEK-293T cells transfected with GFP-TTC36 or si-TTC36 in addition to overexpressing His-Ub and Flag-c-Myc, treated with MG-132 (10 μM) for 6 h, and subjected to immunoprecipitation with Ni-NTA affinity resin followed by immunoblotting with indicated antibodies.

To elucidate the mechanism by which TTC36 promotes HCC, we utilized *Ttc36*^−/−^ mice previously generated in our studies [21]. Remarkably, we observed a significant reduction of c-Myc protein in *Ttc36*^−/−^ mice compared to their WT counterparts (Fig. 2A). Moreover, knockdown of *Ttc36* resulted in down-regulation of c-Myc protein (Figs. 2B, and S2A, Supporting Information), while overexpression of *Ttc36* presented an increase of c-Myc protein in HuH7 and PLC/PRF/5 cells (Fig. 2C), both overexpression and knockdown treatments of *Ttc36* did not alter c-Myc transcription levels (Fig. 2D, E), suggesting that TTC36 affects c-Myc through post-translational mechanisms.

Ubiquitin proteosome system-mediated degradation has been reported in the balance regulation of c-Myc protein [22]. We used CHX to inhibit protein translation or MG-132 to block proteasome function to monitor the turnover of c-Myc. MG-132 treatment reversed the decrease of c-Myc protein induced by knockdown or depletion of *Ttc36* in HuH7 and PLC/PRF/5 (Fig. 2F–I). Elevated levels of *Ttc36* shortened the half-life of c-Myc, while knocking down of *Ttc36* accelerated the degradation of c-Myc protein (Figs. 2J, K, and S2B, C, Supporting Information). Additionally, *Ttc36* overexpression-induced upregulation of c-Myc can also be rescued by simultaneously knocked down of *Ttc36* with specific siRNA (Fig. 2L), and ectopic *Ttc36* expression partially restored *si-c-Myc* induced reduction in protein levels (Figs. 2M, and S2D, Supporting Information). Notably, ubiquitination assays further demonstrated that depletion of *Ttc36* significantly increased c-Myc polyubiquitination, while elevated *Ttc36* expression inhibited c-Myc polyubiquitination (Figs. 2N, and S2E, Supporting Information). These findings provide strong evidence that TTC36 impedes ubiquitin-proteasome system-mediated c-Myc degradation.

### TTC36 Deficiency Enhances Phosphorylation of T58 on c-Myc by GSK3β and Dephosphorylation of S62 on c-Myc

**Fig. 3:**
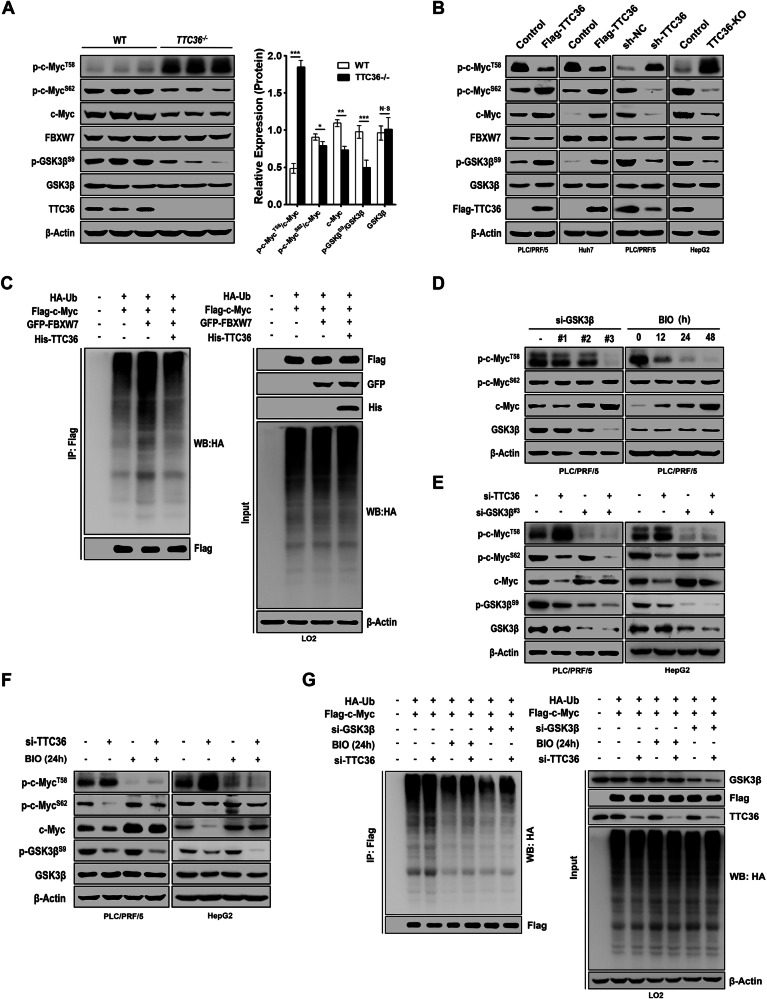
TTC36 inhibits the ubiquitination of c-Myc by FBXW7 by promoting the phosphorylation of GSK3β. **A** Liver cell lysates from wild-type (WT) and TTC36^−/−^ C57BL/6 mice were collected and subjected to immunoblotting with the indicated antibodies. **B** Immunoblotting analysis of PLC/PRF/5 or Huh7 cells overexpressing Flag-TTC36 and PLC/PRF/5 or HepG2 cells with either knockout or knockdown of TTC36. **C** LO2 cells overexpressing GFP-FBXW7 or His-TTC36 in addition to overexpressing HA-Ub and Flag-c-Myc, were treated with MG-132 (10 μM) for 6 h. Cells were harvested with guanidine hydrochloride lysis buffer, immunoprecipitated with 30 μL FLAG affinity beads, and immunoblotted with the indicated antibodies. **D** PLC/PRF/5 cells were transfected with si-GSK3β or treated with the GSK3α/β inhibitor BIO, followed by immunoblotting. **E**, **F** Immunoblotting analysis based on TTC36 knockdown in transfected PLC/PRF/5 cells, GSK3β knockdown with or without GSK3β knockdown, and with or without treatment with BIO. **G** LO2 Cells, in addition to overexpressing His-Ub and Flag-c-Myc, were subjected to various treatment conditions depicted in the figure. In addition, to enhance the detectability of ubiquitinated bands, all samples were treated with MG-132 (10 μM) treatment for 6 h preceded cell harvesting with guanidine hydrochloride lysis buffer. Immunoprecipitation was conducted using 30 μL of FLAG affinity beads, followed by immunoblotting with the indicated antibodies.

Given the ratio of phosphorylation at T58 and S62 by GSK3β on c-Myc determines whether c-Myc enters the degradation cycle [17, 21], we analyzed the phosphorylation of T58 and S62 on c-Myc and the activity of GSK3β in *Ttc36*^−/−^ mice. In *Ttc36*^−/−^ mice, T58 phosphorylation on c-Myc increased significantly while S62 phosphorylation decreased compared to their WT counterparts (Fig. 3A). Additionally, S9 phosphorylation on GSK3β was substantially reduced in *Ttc36*^−/−^ (Fig. 3A). Similar results were obtained for phosphorylation of c-Myc and GSK3β in Ttc36 overexpression in PLC/PRF/5 and HuH7 cells or Ttc36 deficiency in PLC/PRF/5 and HepG2 cells (Fig. 3B). Consistent with publications, high T58 phosphorylation on c-Myc caused the degradation of c-Myc by ubiquitin-proteasome system. Although no direct interaction was detected between *Ttc36* and c-Myc, there is a significant correlation between *Ttc36* expression and GSK3β activity, which influences the phosphorylation and stabilization of c-Myc in HCC and mice models. This implicates that TTC36 might influence c-Myc stability depending on well-known factors. Experiments in HEK-293T and LO2 cells showed that overexpression of *Ttc36* counteracts the ubiquitination (and thus degradation) of c-Myc typically induced by FBXW7 (Figs. 3C, and S3A, Supporting Information). Thus, TTC36 may regulate c-Myc stability through established pathways, potentially impeding FBXW7-mediated polyubiquitination and degradation of c-Myc via T58 phosphorylation.

**Fig. 4:**
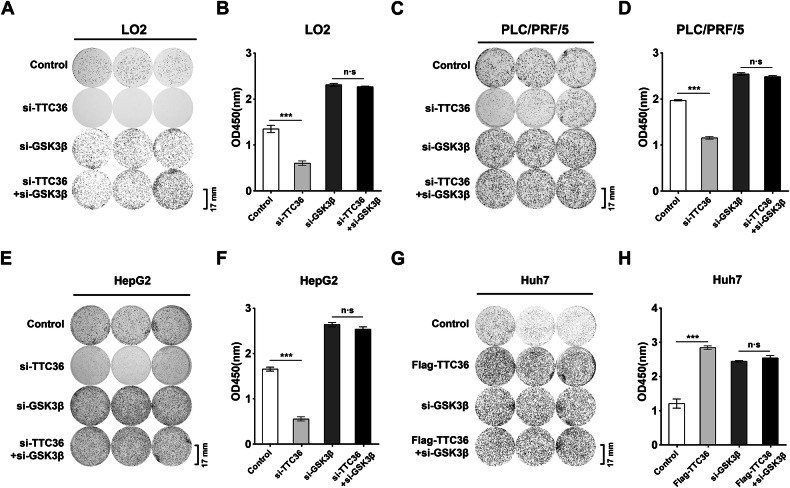
Tumor-proliferating effects of TTC36 depletion are GSK3β-dependent. **A**, **C**, **E**, **G** A colony formation assay was used to detect whether si-GSK3β would hinder the growth-promoting effect of si-TTC36 inhibition or overexpression-induced normal human hepatocytes or hepatoma cells. **B**, **D**, **F**, **H** The effects of si-TTC36 inhibition or overexpression on the in vitro growth of normal human hepatocytes or hepatocellular carcinoma cells were evaluated. Potential blocking of this inhibition by si-GSK3β was evaluated using Cell Counting Kit-8.

GSK3β is recognized as the kinase responsible for phosphorylating T58 on c-Myc. To further validate the stabilization of c-Myc promoted by TTC36, depending on GSK3β-FBXW7 signaling axis, *si-GSK3β* or BIO was utilized to inhibit GSK3β phosphorylation of T58 on c-Myc. Both *si-GSK3β* and BIO treatments resulted in decreased phosphorylation of T58 on c-Myc, thereby stabilizing c-Myc protein (Fig. 3D). Meanwhile, neither treatment had any effect on the phosphorylation of S62 on c-Myc (Fig. 3D). Next, we used si-GSK3β or BIO (a kinase inhibitor of GSK3β) to block the function of GSK3β. The changes in c-Myc and phosphorylation of T58 caused by TTC36 knockdown in PLC/PRF/5 and HepG2 cells were blocked by inhibition of GSK3β. Notably, the phosphorylation of S62 caused by TTC36 knockdown was not disturbed (Fig. 3E, F). Consistent with these findings, ubiquitin-modified c-Myc induced by *TTC36* deficiency was also blocked under *si-GSK3β* or BIO treatment (Figs. 3G and S3B, Supporting Information). Additionally, the treatments of *si-GSK3β* abrogated the proliferation of LO2, PLC/PRF/5, HepG2, and Huh7 cells induced by TTC36(Fig. 4A–I). These findings indicated TTC36 decreased GSK3β-phosphorylated-T58 on c-Myc.

### TTC36 Reduces PP2A Activity by Facilitating the Binding of SET to PPP2R1A

**Fig. 5:**
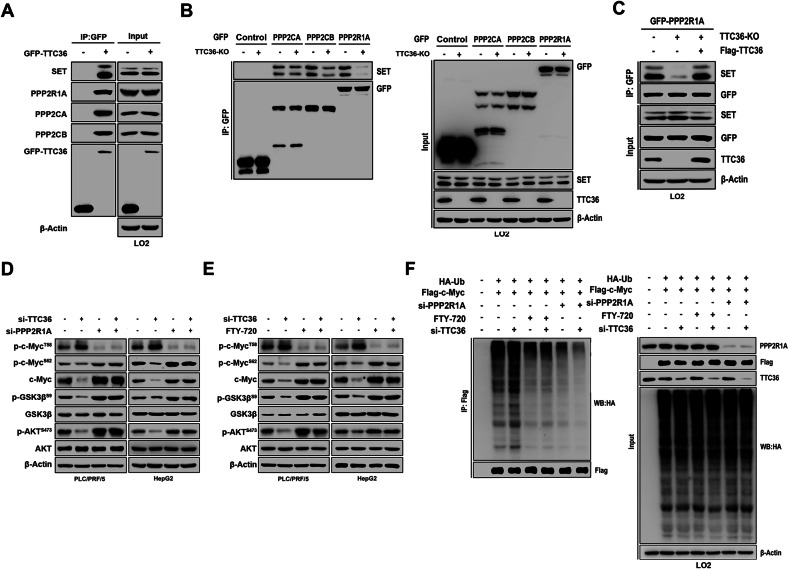
TTC36 depletion destabilizes c-Myc by attenuating the interaction between SET and PPP2R1A. **A** LO2 cell was transfected with the specified plasmids, and GFP magnetic beads facilitated immunoprecipitation. Subsequent immunoblotting analysis utilized the respective primary antibodies. **B**, **C** LO2 cells with or without TTC36 knockout underwent treatment according to indicated conditions, followed by immunoblotting analysis with corresponding primary antibodies after Immunoprecipitation using GFP magnetic beads. **D**, **E** Based on the knockdown of TTC36 transfected in PLC/PRF/5 cells and HepG2 cells, PP2A inhibitor or si-PPP2R1A was used to observe changes in downstream target proteins. Western blot analysis was performed using the corresponding antibodies in the figure, with β-Actin protein as an internal control. **F** LO2 cell, concomitantly overexpressing His-Ub and Flag-c-Myc, experienced diverse treatment conditions as illustrated in the figure. Preceding cell harvesting with guanidine hydrochloride lysis buffer, immunoprecipitation was performed using 30 μL of FLAG affinity beads, followed by immunoblotting with the indicated antibodies.

PP2A acts as a phosphatase, dephosphorylating c-Myc (S62) while also dephosphorylating GSK3β (S9) and AKT (S473) simultaneously [23, 24]. To investigate the underlying mechanisms of phosphorylation changes induced by TTC36 deficiency on c-Myc, GSK3β, and AKT, we analyzed the interaction between TTC36 and the PP2A complex. Co-immunoprecipitation assays in HEK-293T cells confirmed the interaction between PPP2R1A or SET and TTC36, which was further validated by *Ttc36* overexpression in LO2 and HEK-293T cells (Figs. 5A, and S4A–S4B). SET interacts with PP2AC or PPP2R1A, inhibiting PP2A activity [25]. TTC36 deficiency disrupted this interaction, particularly affecting SET-PPP2R1A but not SET’s interactions with PP2ACA and PP2ACB (Figs. 5B and S4C, Supporting Information), and this disruption was reversed by *Ttc36* overexpression (Figs. 5C, and S4D, Supporting Information). Knocking down of *PPP2R1A* or FTY-720 (PP2A inhibitor) treatment increased the phosphorylation of S9 at GSK3β and S473 at AKT (lane 3 compared with lane 1), countering the impact of si-*Ttc36* on the phosphorylation of S62 and T58 at c-Myc (Figs. 5D, E, and S4E, Supporting Information). Inhibition of PP2A also prevents c-Myc polyubiquitination induced by *Ttc36* knockdown (Figs. 5F, and S4F, Supporting Information). This indicates that TTC36 reduced the phosphorylation of S62 at c-Myc through inhibition of PP2A activity, indirectly influencing p-c-Myc^T58^ via the PP2A/AKT/GSK3β pathway. Consequently, this modulation results in the failure of c-Myc to undergo degradation.

### Phosphorylation of S125 at TTC36 stabilizes the c-Myc protein, leading to sorafenib resistance

**Fig. 6:**
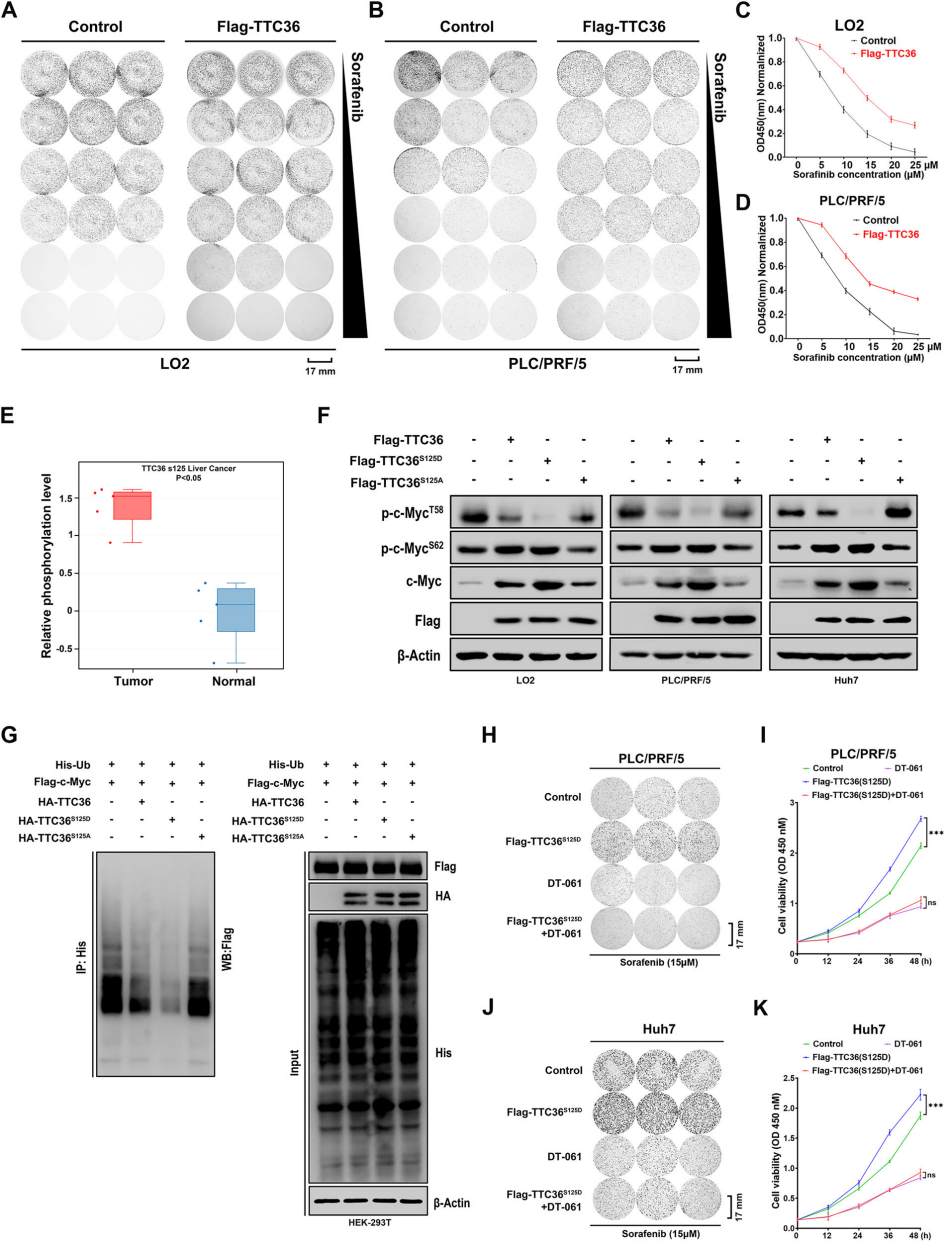
TTC36^S125D^ but not TTC36^S125A^ exerts its oncogenic role in HCC. **A**, **B** LO2 or PLC/PRF/5 cells with Flag-TTC36 were subjected to Colony Formation assay, sorafenib concentration gradient was 0, 5, 10, 15, 20, 25, and the same volume of DMSO was used as a blank control. **C**, **D** In vitro growth of LO2 or PLC/PRF/5 cells treated with R of Flag-TTC36 by Cell Counting Kit-8. **E** Analysis of TTC36 phosphorylation levels in the TCGA-LIHC cohort. **F** The target plasmid was transfected into LO2, PLC/PRF/5, and Huh7 cells as shown in the figure, and then Western blotting was performed using the corresponding antibodies in the figure. **G** HEK-293T cells were transfected with plasmids as shown in the figure, treated with MG-132 (10 μM) for 6 h before cell collection with Guanidine hydrochloride lysis buffer, immunoprecipitated with Ni-NTA affinity resin, and immunoblotted with specified antibodies. **H**, **J** Colony formation assay of PLC/PRF/5 or Huh7 under the conditions illustrated. **I**, **K** Evaluation of in vitro growth of PLC/PRF/5 or Huh7 cells under the conditions depicted in the figure using the Cell Counting Kit-8.

Overexpression of *Ttc36* in LO2 and PLC/PRF/5 cells exhibits resistance to sorafenib, supported by cellular cloning and viability assays (Fig. 6A–D). Upon scrutinizing the phosphorylation data from the TCGA-LIHC project, an aberrant phosphorylation pattern emerged at the S125 site of TTC36 in HCC (Fig. 6E). These observations lead us to hypothesize that phosphorylation at S125 on TTC36 may play a role in its pro-carcinogenic function. To validate this hypothesis, TTC36 mutants, TTC36^S125D^ and TTC36^S125A^, were constructed to mimic phosphorylation activation and inactivation states, respectively. Notably, TTC36^S125D^, but not TTC36^S125A^, suppressed the phosphorylation of T58 on c-Myc while enhancing the phosphorylation of S62 on c-Myc (Fig. 6F), consequently reducing c-Myc degradation (Figs. 6G, and S5A, Supporting Information). Meanwhile, compared with other groups, TTC36S125D caused the strongest sorafenib resistance in PLC/PRF/5, Huh7, and LO2 cells (Fig. S5B–D, Supporting Information). Furthermore, the sorafenib resistance induced by TTC36^S125D^ was effectively reversed by DT-061 (PP2A activator) (Figs. 6H–K, and S5E, F, Supporting Information). These findings indicate that phosphorylation at S125 of TTC36 contributes to the prevention of c-Myc degradation and promotes HCC development.

### TTC36 promotes cell proliferation and tumor formation in a c-Myc-dependent manner

**Fig. 7:**
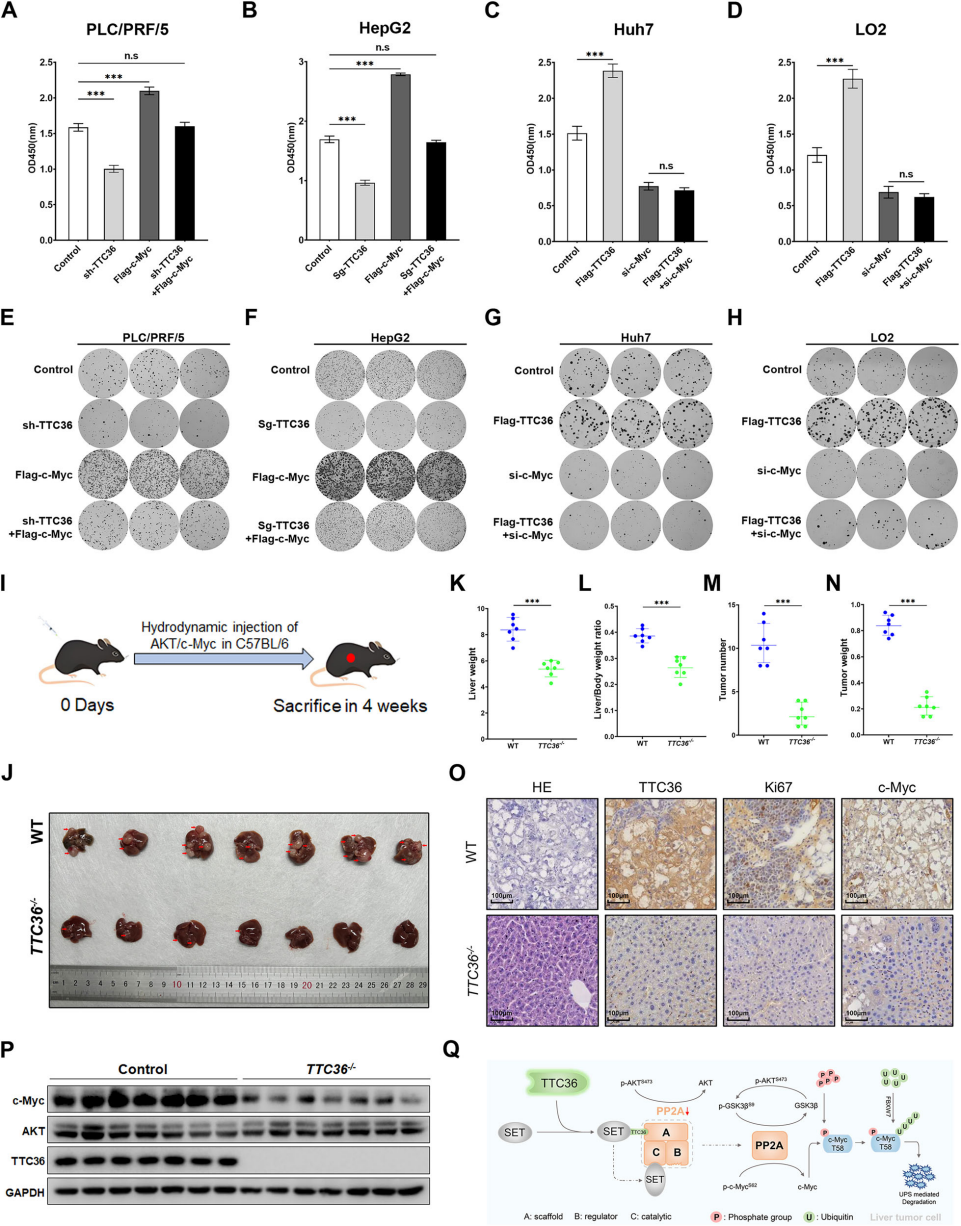
The oncogenic effect of TTC36 is dependent on c-Myc in HCC. **A**, **B** The effects of si-TTC36 inhibition or knockout on the in vitro growth of hepatocellular carcinoma cells were evaluated. Potential blocking of this inhibition by Flag-c-Myc was evaluated using Cell Counting Kit-8. **C**, **D** The effects of TTC36 overexpression on the in vitro growth of Huh7 or LO2 cells were evaluated. Potential blocking of this inhibition by si-c-Myc was evaluated using Cell Counting Kit-8. **E**, **F** A colony formation assay was used to detect whether Flag-c-Myc would hinder the growth-promoting effect of TTC36 inhibition or knockout-induced hepatoma cells. **G**, **H** A colony formation assay was used to detect whether si-c-Myc would hinder the growth-promoting effect of TTC36 overexpression-induced hepatoma cells. **I** Hydrodynamic injection of HCC original cancer mouse model. **J** Gross liver anatomy and tumor formation in wild-type and TTC36 knockout mice (4 weeks after injection). Liver weight (**K**), Liver/Body weight ratio (**L**), Tumor number(**M**), Tumor weight (**N**) of adult WT, and *TTC36*^*−/−*^ mice (*n* = 7; mean ± SD). **O** Representative images of H&E, TTC36, Ki67, and c-Myc immunostaining of wild-type and TTC36 knockout mouse liver sections. **P** Immunoblotting of liver lysates from wild-type (WT) and TTC36^−/−^ C57BL/6 mice using the antibodies shown in the figure; GADPH served as the loading control (*N* = 7). **Q** Work model.

To investigate whether TTC36 regulates HCC progression through c-Myc, we used siRNA to inhibit c-Myc expression. The results showed that the decreased cell proliferation caused by TTC36 knockdown or knockout could be rescued and blocked by c-Myc overexpression (Figs. 7A–H, and S6A–C, Supporting Information). Specifically, CCK-8 cell proliferation assays revealed that TTC36 inhibition or knockout significantly suppressed the proliferation of PLC/PRF/5 and HepG2 cells, an effect reversed by Flag-c-Myc (Fig. 7A, B). Similarly, TTC36 overexpression enhanced the proliferation of Huh7, LO2, PLC/PRF/5, and HepG2 cells, which was also blocked by si-c-Myc (Figs. 7C, D, and S6A, B, Supporting Information). These findings were further supported by colony formation assays, which confirmed that TTC36-dependent proliferation promotion in HCC (Figs. 7E–H, and S6C, D, Supporting Information). Additionally, an orthotopic liver cancer model was established using hydrodynamic injection of AKT/c-Myc plasmids, and tumor samples were collected four weeks post-injection (Fig. 7I). Compared to the control group, TTC36-knockout mice exhibited significantly reduced tumor formation, lower liver weight, and diminished tumor burden (Fig. 7J–N). Immunohistochemistry and Western blot analyses confirmed the loss of TTC36 and c-Myc downregulation in knockout mice (Fig. 7O, P). Histologically, TTC36-knockout mice displayed smaller tumor necrotic areas, fewer proliferating cells (as indicated by reduced Ki-67 immunoreactivity), and impaired tumor growth (Fig. 7O). These results suggest that the absence of TTC36 suppresses tumor cell proliferation, with reduced c-Myc expression serving as a critical mechanism underlying the observed inhibition of HCC progression.

## Discussion

This study unveils a novel role for TTC36 in orchestrating c-Myc protein stability, a pivotal determinant in the emergence of sorafenib resistance in HCC. Our investigations reveal that TTC36 facilitates SET-mediated inhibition of PP2A activity, thereby reinforcing c-Myc stability through the AKT/GSK3β axis. These intricate regulatory pathways amplify the proliferative, migratory, and sorafenib-resistant of HCC cells. Notably, TTC36 emerges as a hitherto unrecognized regulatory player in the SET/PP2A/c-Myc axis, underscoring its potential as a promising therapeutic target for HCC intervention.

TTC36, previously identified in our murine model, was found to be closely associated with tyrosinemia [19]. Comprehensive assessments encompassing proliferation, migration, cell cycle, apoptosis, and sorafenib resistance of HCC cells, we observed that overexpression of *Ttc36* significantly performed as a tumor-promoting factor from the phenotypes, while knockdown or deletion of *Ttc36* exerted inhibitory effects. However, previous studies have reported that silencing TTC36 may lead to overexpression of Wnt/β-catenin signaling components [26]. To resolve this conflict and provide new insights about TTC36 in the development of sorafenib resistance during HCC treatment [14, 15, 27]. We examined c-Myc protein levels in *Ttc36*^−/−^ mice [21], revealing lower c-Myc protein levels in these mice, consistent at the cellular level. Further investigations unveiled that TTC36 regulates c-Myc protein stability through the ubiquitin-proteasome system, influencing the occurrence of stemness and resistance in HCC [28, 29]. Although our results suggest that TTC36 promotes tumor proliferation in a c-Myc-dependent manner, direct evidence linking TTC36 silencing to activation of the Wnt/β-catenin pathway is lacking in this study. Based on our knowledge, this is the first insight about TTC36 in the regulation of c-Myc, which highlights the complexity of targeting TTC36 in a therapeutic setting.

FBXW7 acts as a crucial E3 ligase in the ubiquitin-proteasome pathway, orchestrating c-Myc degradation. Earlier studies emphasized GSK3β‘s role in phosphorylating c-Myc’s T58 site (p-c-MycT58), vital for FBXW7-mediated c-Myc degradation [17]. In *Ttc36*^−/−^ mice, GSK3β and FBXW7 levels remained unchanged, but p-GSK3βS9 and p-c-MycS62 notably decreased. Overexpressing *Ttc36* in vitro hindered FBXW7-mediated c-Myc polyubiquitination, a process mitigated by si-*GSK3β* or BIO (a GSK3β inhibitor). This blockade impeded TTC36’s impact on HCC cell proliferation and c-Myc stability without affecting p-c-MycS62 downregulation. TTC36 appears to modulate c-Myc protein stability by altering c-Myc phosphorylation.

These findings led us to explore p-GSK3βS9 and p-c-MycS62, both PP2A dephosphorylation targets [30, 31], with p-GSK3βS9 additionally activated by AKT [32]. PP2A, comprising subunits A, B, and C (two isoforms, α and β) [33], faces inhibition by SET, interacting with PPP2R1A and PP2ACA [25, 34]. Immunoprecipitation experiments confirmed that TTC36 deficiency primarily disrupts SET and PPP2R1A interaction, liberating PP2A activity. Subsequent rescue experiments revealed that inhibiting PP2A activity could rescue TTC36’s impact on c-Myc phosphorylation, polyubiquitination, and sorafenib resistance observed with *Ttc36* overexpression. This unveils a novel avenue for regulating PP2A activity, presenting promising therapeutic possibilities for patients with c-Myc amplification and sorafenib resistance.

## Conclusion

This study elucidates the critical regulatory mechanism of TTC36 in proliferation and sorafenib resistance in HCC cells. By influencing SET to inhibit PP2A activity, TTC36 stabilizes the c-Myc protein, thereby promoting the proliferation, migration, and sorafenib resistance of liver cancer cells. The phosphorylation status of serine 125 in TTC36 significantly impacts the phosphorylation and stability of c-Myc, particularly in the ubiquitination and degradation pathways. These findings provide new insights into the development of therapeutic strategies targeting TTC36, revealing the potential of inhibiting PP2A activity in treating patients with c-Myc amplification and sorafenib resistance.

## Materials and methods

### Cell lines and chemical reagents

HEK-293T, LO-2, PLC/PRF/5, HepG2, and Huh7 cell lines were obtained from ATCC (Manassas, Virginia). Cells were cultured in a medium supplemented with 10% fetal bovine serum (FBS) and dual antibiotics (penicillin at 100 U/ml and streptomycin at 100 U/ml) at 37 °C in a humidified incubator. Detailed information on all chemical reagents and antibodies is provided in Supplementary Table 1.

### DNA constructs and mutagenesis

Human *Ttc36* (NM_001080441.2), *c-Myc* (NM_001354870.1), *FBXW7* (NM_001013415.2), *PPP2CA* (NM_001355019.2), *PPP2CB* (NM_001009552.2), *SET* (NM_001122821.2), and ubiquitin (NM_001281716.1) were PCR-amplified and subcloned into CMV, pTriEX, or pcDNA3.1 vectors. Gene knockdown or knockout plasmids were constructed using Lenti V2 and shRNA pGIPZ vectors. Additionally, targeted mutations in *Ttc36* were introduced using PCR amplification. Primer sequences and detailed information for all DNA plasmid constructs are provided in Supplementary Tables 2–5.

The procedures for isolating and culturing primary mouse hepatocytes, lentiviral infection, establishing stable cell lines, RNA extraction, q-PCR, Immunoprecipitation, and immunoblotting were performed following established protocols as described in previous studies [19].

### Human samples and immunohistochemical staining

Tissue samples were obtained from two HCC patients who underwent liver resection prior to chemotherapy at the First Affiliated Hospital of Chongqing Medical University. Written informed consent for tumor analysis was obtained from both patients. Eight paraffin-embedded tissue samples (Four paracancerous and four tumor tissues) were serially sectioned into 4 μm slices. Hematoxylin and eosin (H&E) staining and immunohistochemical (IHC) staining were performed on the sections. IHC was conducted using a polyclonal antibody against TTC36, produced in-house, at a dilution ratio of 1:100. The avidin-biotin-peroxidase complex method was applied for visualization. Stained tissue sections were digitally scanned using a Leica DM6B microscope to capture high-resolution images. The study protocol was reviewed and approved by the Ethics Committee of the First Affiliated Hospital of Chongqing Medical University.

### Animal experiments, primary hepatocyte extraction, and tail vein plasmid injection

Ttc36^−/−^ C57BL/6 mice, originally developed and generously provided by Professor Zhouqin in 2014, were maintained under standard laboratory conditions (12:12-h light-dark cycle, lights on at 07:00 A.M., 23 °C temperature, 40–50% relative humidity) with ad libitum access to food and water [21]. Primary hepatocyte extraction was performed following our previously established protocol, while the tail vein plasmid injection method was slightly modified from prior procedures [19]. Specifically, the plasmid solution was prepared in normal saline, with 2 mL of solution injected per 20 g of body weight via the tail vein within 5–8 s. After plasmid delivery, the mice were housed until tumor formation. All surgical procedures were conducted under sodium pentobarbital anesthesia, and postoperative care was administered in compliance with the Guidelines for the Management and Use of Laboratory Animals of Chongqing Medical University. The experimental protocols were approved by the Laboratory Animal Ethics Committee of Chongqing Medical University.

### Ubiquitination assay

HEK-293T or LO-2 cells were co-transfected with respective plasmids, as indicated in the corresponding figure legends. A 6 M concentration of HCl-guanidine solution was utilized to lyse cells, and 10 μM MG-132 was added 6 h before protein collection to prevent deubiquitination. Ni-NTA resin or Flag magnetic beads were separately employed for His-Ub or Flag-c-Myc affinity binding. The cell lysate was incubated with beads at 4 °C for 12 h. Subsequently, beads were washed thrice with lysis buffer, and immunoblotting with anti-Flag antibody (Flag-c-Myc) or HA antibody (HA-Ub) was performed to detect the ubiquitination status. Finally, internal protein expression, such as β-actin, was assessed using additional antibodies, serving as loading controls.

### Cell proliferation assays

Cell proliferation was assessed using CCK-8 and colony formation assays. For the CCK-8 assay, LO2, PLC/PRF/5, Huh7, or HepG2 cells were seeded in a 96-well plate at a density of 1500 cells per well. Following the manufacturer’s instructions, optical density at 450 nm (OD450) was measured at 0, 24, 48, and 72 h after seeding. Colony formation assays involved seeding transfected cells at a density of 600 cells per well in 6-well plates and maintaining them for 8 days in the culture medium. Colonies were fixed, stained with 0.2% crystal violet, photographed, and subsequently counted.

### Real-time continuous assessment of cellular growth

Cellular growth in cell lines expressing *Ttc36* or *Ttc36* siRNA was monitored using the xCELLigence RTCA DP system (ACEA Biosciences Inc., San Diego, CA). A specially designed 16-well plate with a gold-coated bottom was utilized for seeding 2500 cells. After incubating at room temperature for a minimum of 20 min, cells were placed in the RTCA DP system incubator. Data were automatically collected every 15 min by the analyzer, controlled by integrated software.

### MTT Assay

PLC/PRF/5 cells in the exponential growth phase were digested and seeded into a 96-well microplate at a density of 2 × 10^3^ cells per well with 100 µl of complete culture medium. The cells were then incubated at 37 °C with 5% CO_2_. At 24, 48, or 72 h, the medium was replaced with 100 µl of MTT solution (0.5 mg/ml) and further incubated at 37 °C for 4 h. The resulting crystals were dissolved in 150 µl of DMSO, and the absorbance at 490 nm was recorded to assess formazan formation.

### Wound healing assay

PLC/PRF/5 cells were cultured in 6-well plates until reaching 95%–100% confluence. Scratch wounds were generated using a 200 µl pipette tip, followed by two washes with phosphate-buffered saline (PBS) (pH 7.4) to eliminate cell debris. The cells were then incubated in complete culture medium at 37 °C with 5% CO_2_. Fluorescence microscope images of wound width were captured at three time points (0, 12, and 24 h). Six photographs were taken from independently selected fields for each sample, and the width of the wound areas was calculated.

### Flow cytometry assay

PLC/PRF/5 cells overexpressing *Ttc36* or *Ttc36* shRNA were harvested with EDTA-free trypsin when reaching 80%–90% confluence. Apoptosis was assessed using the Annexin V-fluorescein isothiocyanate (FITC) Apoptosis Detection Kit (KeyGEN BioTECH, Nanjing, China). For cell cycle analysis, cells were fixed with pre-cooled 70% ethanol at −20 °C, resuspended in 1 mL PI/Triton X-100 staining solution (20 μg PI/0.1% Triton X-100) containing 0.2 mg RNase A, and stained for 15 min at 37 °C. Single cells were then analyzed by flow cytometer according to the manufacturer’s instructions. The apoptosis and cell cycle analyses were conducted by the College of Life Sciences at Chongqing Medical University.

### Immunoprecipitation and immunoblotting analysis

The immunoprecipitation and immunoblotting analyses followed the previous research [19]. Proteins were extracted from cultured HEK-293T cells, LO2 cells, PLC/PRF/5cells, HepG2 cells, Huh7 cells, and mouse primary hepatocytes using a lysis buffer consisting of 50 mM Tris–HCl pH 7.5, 0.1% SDS, 1% Triton X-100, 150 mM NaCl, 1 mM dithiothreitol, 0.5 mM EDTA, 100 mM PMSF, 100 mM leupeptin, 1 mM aprotinin, 100 mM sodium orthovanadate, 100 mM sodium pyrophosphate, and 1 mM sodium fluoride. The cell extracts were clarified by centrifugation at 13,000 × *g*, and the resulting supernatants (2 mg protein mL^−1^) underwent immunoprecipitation using the specified antibodies. Following overnight incubation at 4 °C, protein A agarose beads were added and incubated for an additional 3 h. The immunocomplexes were washed three times with lysis buffer and then subjected to immunoblotting analyses using corresponding antibodies and supplementary Table 1. Band intensities were quantified using the Image Lab software program (Bio-Rad).

### Immunohistochemical analysis

Mice were euthanized via CO_2_ inhalation, and liver tissues were collected, paraffin-embedded, and stained with hematoxylin and eosin (H&E). IHC staining was performed following standard protocols. Briefly, tissue sections were deparaffinized, rehydrated through a graded ethanol series, and subjected to antigen retrieval using sodium citrate or Tris-EDTA buffer, as specified by the antibody manufacturer. Sections were blocked with 10% FBS in PBS for 30 min at room temperature. To inhibit endogenous peroxidase activity, sections were treated with 3% hydrogen peroxide in methanol for 10 min at room temperature. Samples were then incubated with primary antibodies, followed by detection with horseradish peroxidase (HRP)-conjugated secondary antibodies using the DAB detection method. Images of stained slides were captured using a Widefield Zeiss Observer Seven microscope.

### Data analysis and statistics

All experiments were conducted at least three times, and results are presented as Mean ± SD. Statistical analysis was performed using Prism software (GraphPad Software). *P* values were determined using two-tailed Student’s *t*-tests, with significance indicated as follows: *P ≤ 0.05; **P ≤ 0.01; ***P ≤ 0.001; ****P ≤ 0.0001.

## Supplementary information

Supplementary information

Raw WB

## Data Availability

All data are available in the main text or the supplementary materials.
