# Correction: TTC36 promotes proliferation and drug resistance in hepatocellular carcinoma cells by inhibiting c-Myc degradation

**DOI:** 10.1038/s41419-025-08407-0

**Published:** 2026-01-28

**Authors:** Fengling Shao, Runzhi Wang, Xinyi Li, Yanxia Hu, Zaikuan Zhang, Jing Cai, Jieru Yang, Xiaosong Feng, Suxia Ren, Zengyi Huang, Yajun Xie

**Affiliations:** 1 The Ministry of Education Key Laboratory of Laboratory Medical Diagnostics, the College of Laboratory Medicine, Chongqing Medical University, Chongqing, China; 2 School of Life and Health Sciences, Hainan University, Haikou, China; 3 College of Basic Medical Sciences, Harbin Medical University, Harbin, China; 4 Department of Cell Biology and Genetics, School of Basic Medical Sciences, Chongqing Medical University, Chongqing, China; 5 Mitomedical laboratory of Children’s Hospital of Chongqing Medical University, National Clinical Research Center for Child Health and Disorders, Ministry of Education Key Laboratory of Child Development and Disorders, Chongqing Key Laboratory of Child Rare Diseases in Infection and Immunity, Chongqing, China

**Keywords:** Molecular biology, Gastrointestinal cancer

Correction to: *Cell Death & Disease* 10.1038/s41419-025-07663-4, published online 24 April 2025

We identified an inadvertent error in Figure 4E (colony formation assay), where an incorrect image was used (see attached Errata Image Description). This appears to have occurred during image cropping and pasting, and was not intentional. We retrieved the original raw images (attached as Raw Image) to confirm the integrity of our data.

The specific reason: We used Photoshop to crop the original image and then copied and pasted it. Unfortunately, during the copy-paste process, part of the image was additionally copied and placed at the top layer, causing the original image to be obscured by the extra copy. In other words, the true clone-formed image resided at the bottom layer, which was obscured by the extra copy, resulting in the final output image appearing as if the image had been reused.

**Original Figure 4** 
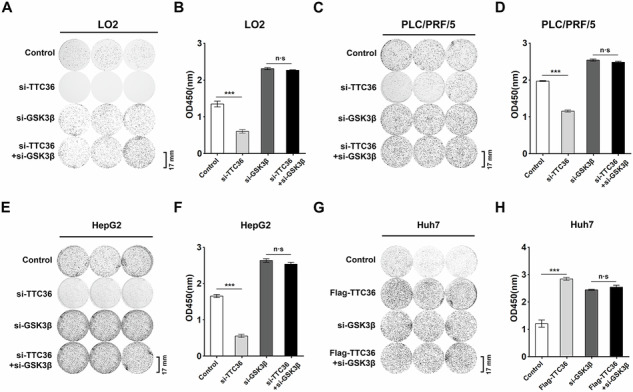


**Amended Figure 4** 
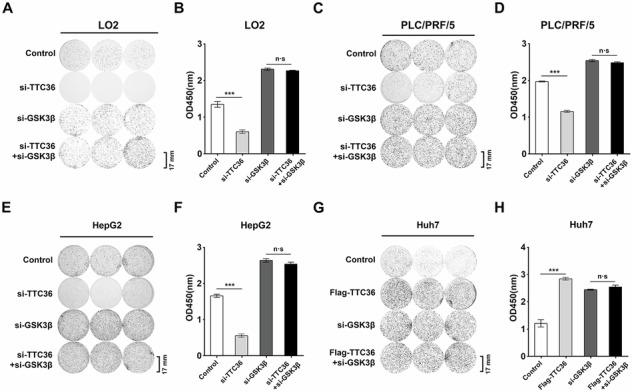


The original article has been corrected.

## Supplementary information

Original data_Figure 4

