# Supplementary material for: TTC36 promotes proliferation and drug resistance in hepatocellular carcinoma cells by inhibiting c-Myc degradation
Source: Cell Death Dis. 2025 Apr 24;16(1):332. doi: 10.1038/s41419-025-07663-4 (PMC12022016; doi:10.1038/s41419-025-07663-4)
Supplement: Supplementary file 1 — Supplementary information [file 41419_2025_7663_MOESM1_ESM.pdf]

Supplementary information

TTC36 promotes proliferation and drug resistance in hepatocellular carcinoma cells by inhibiting c-Myc degradation

Fengling Shao[1, †], Runzhi Wang[1, †], Xinyi Li[1], Yanxia Hu[2], Zaikuan Zhang[1], Jing Cai[3], Jieru Yang[1], Xiaosong Feng[1], Suxia Ren[4*], Zengyi Huang[4, 5, *] and Yajun Xie[1, *]

Supplementary Figures

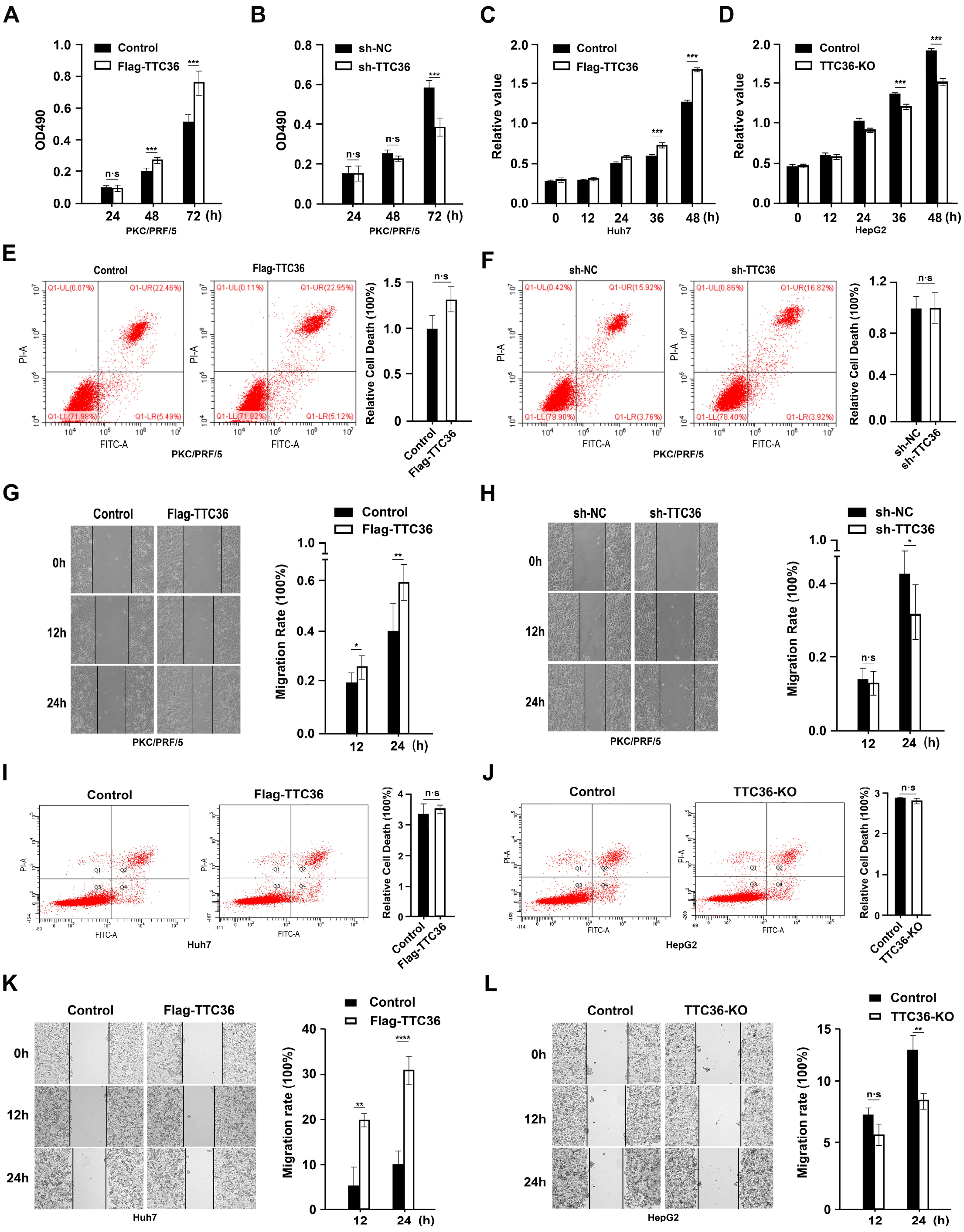

Figure S1. TTC36 Promotes cell proliferation and induces migration and cell cycle of hepatocellular carcinoma Cells.

(A-B) The growth of PLC/PRF/5 cells transfected with Flag-TTC36 or si-TTC36 was determined using the MTT assay. Results are presented with error bars indicating mean ± SD (n = 6). ***p < 0.001.

(C-D) In vitro growth of PLC/PRF/5 cells treated with R of Flag-TTC36 or sh-TTC36 assessed by Cell Counting Kit-8.

(E-F) Apoptosis of PLC/PRF/5 cells was determined by flow cytometry with Annexin V-FITC/propidium iodide staining. The corresponding quantitative analysis is shown on the right (mean ± SD (n = 3)).

(G-H) Cell migration of PLC/PRF/5 cells was assessed using the Wound Healing assay, with the width of the wound area calculated at indicated time points. Results are presented with error bars indicating mean ± SD (n = 6). $^{*}p < 0.05$, $^{**}p < 0.01$.

(I-J) Apoptosis in Huh7 or HepG2 cells assessed by flow cytometry and Annexin V-FITC/propidium iodide staining, with corresponding quantitative analyses on the right (mean ± SD (n = 3)).

(K-L) Cell migration of Huh7 or HepG2 cells evaluated by the wound healing assay, with the width of the wound area calculated at the indicated time points. Results are shown with error bars representing mean ± SD (n = 6). $^{***}p<0.001$.

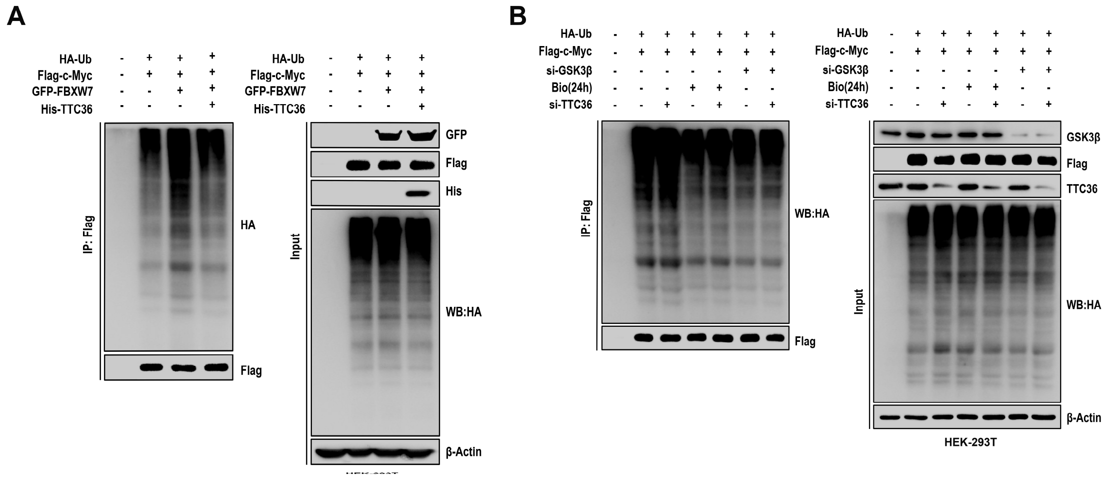

Figure S2. TTC36 regulates c-Myc stability through the ubiquitin proteasome system.

(A) Q-PCR analysis of TTC36 and c-Myc mRNA levels in HepG2, normalized to 18S, with error bars representing mean ± SD (n = 3). ***p<0.001.

(B, C) Protein blot analysis of Huh7 cells overexpressing Flag-TTC36 or TTC36-depleted HepG2 cells, treated or untreated with CHX at specified time points.

(D) Protein blot analysis of si-c-Myc knockdown efficiency in PLC/PRF/5 cell, with β-actin as the loading control.

(E) Co-transfection of HEK-293T cells with Flag-c-Myc and His-Ub, followed by transfection or non-transfection of GFP-TTC36 or si-TTC36. Cells were treated with MG-132 (10 μM) for 6 hours before harvesting with guanidine hydrochloride lysis buffer. Immunoprecipitation was performed using Ni-NTA affinity resin, followed by immunoblotting with the specified antibodies.

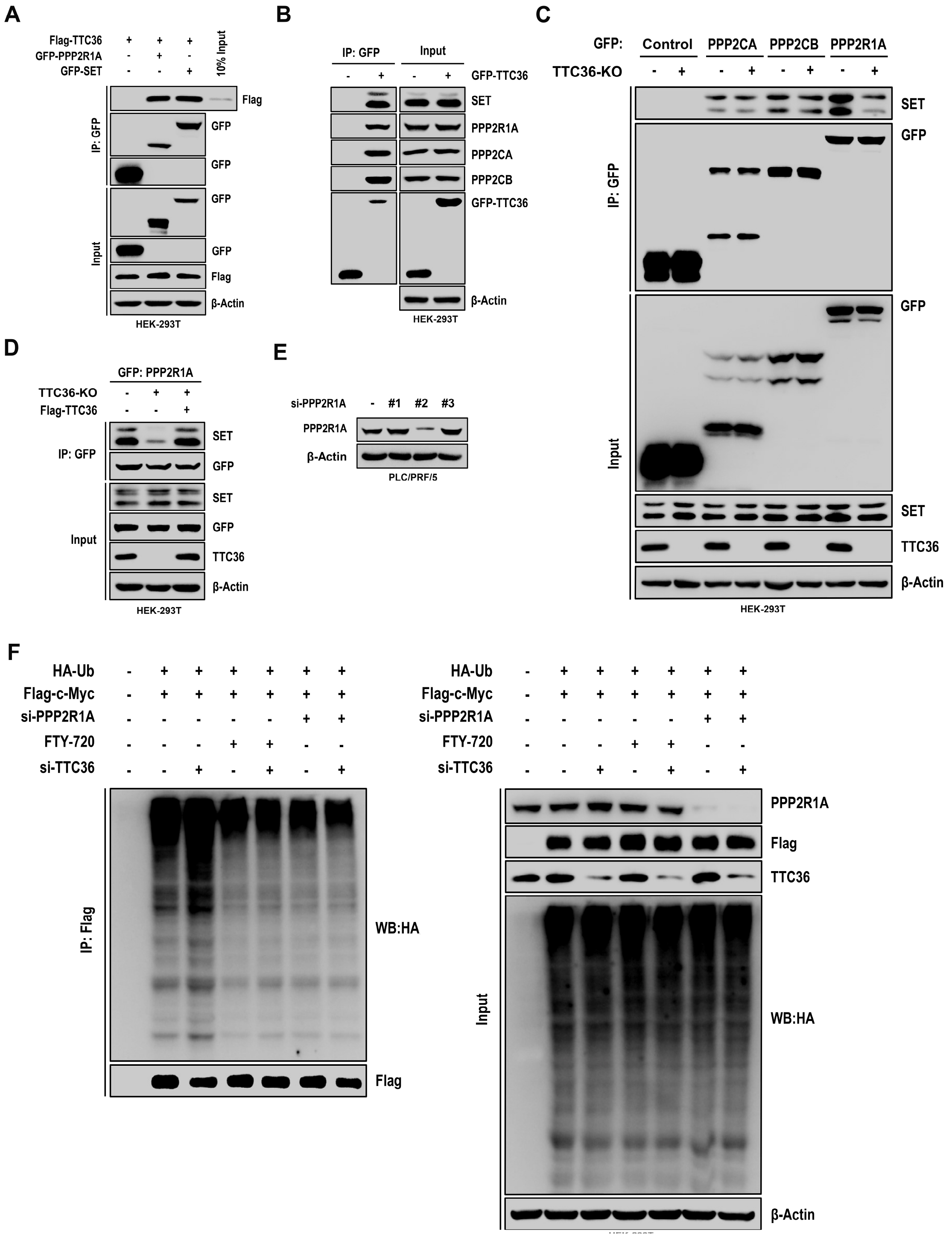

Figure S3. TTC36 inhibits c-Myc polyubiquitination promoted by FBXW7 and dependent on GSK3β.

(A) HEK-293T cells overexpressing GFP-Fbxw7 or His-TTC36 in addition to overexpressing HA-Ub and Flag-c-Myc, were treated with MG-132 (10 μM) for 6 hours. Cells were harvested with guanidine hydrochloride lysis buffer,

immunoprecipitated with 30 μL FLAG affinity beads, and immunoblotted with the indicated antibodies.

(B) HEK-293T cells, additionally overexpressing HA-Ub and Flag-c-Myc, underwent various treatments as illustrated in the figure. Before cell harvesting with guanidine hydrochloride lysis buffer, MG-132 (10 μM) treatment for 6 hours was applied. Immunoprecipitation was performed using 30 μL of FLAG affinity beads, followed by immunoblotting with the indicated antibodies.

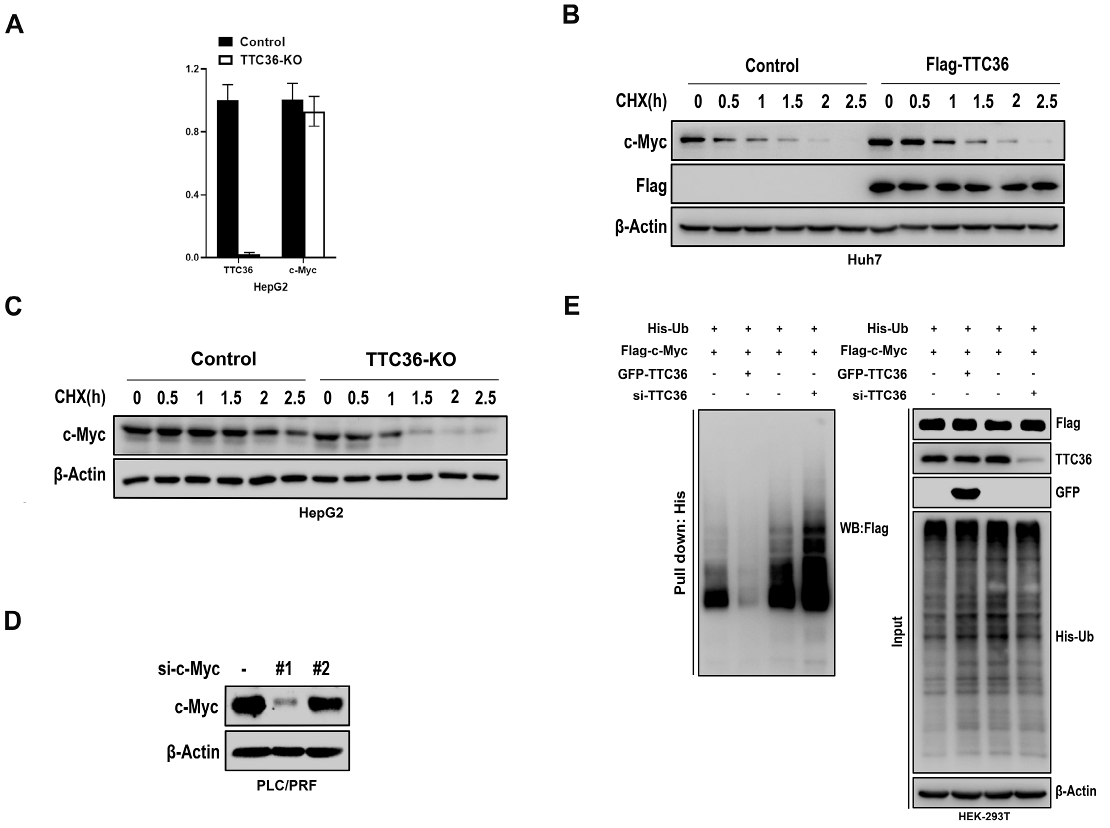

Figure S4. TTC36 depletion weakens the interaction between SET and PPP2R1A thereby promoting c-Myc polyubiquitination.

(A-B) HEK-293T cell were transfected with the specified plasmids, and GFP magnetic beads facilitated immunoprecipitation. Subsequent immunoblotting analysis utilized the respective primary antibodies.

(C-D) HEK-293T cells with or without TTC36 knockout underwent treatment according to indicated conditions, followed by immunoblotting analysis with corresponding primary antibodies after Immunoprecipitation using GFP magnetic beads.

(E) Verification of si-PPP2R1A knockdown efficiency via protein blot analysis in PLC/PRF/5 cells, with β-actin serving as the loading control.

(F) HEK-293T cells, concurrently overexpressing HA-Ub and Flag-c-Myc, underwent diverse treatment conditions as depicted. Immunoprecipitation was conducted using 30 μL FLAG affinity beads before cell harvest with guanidine hydrochloride lysis buffer, followed by immunoblotting with the indicated antibodies.

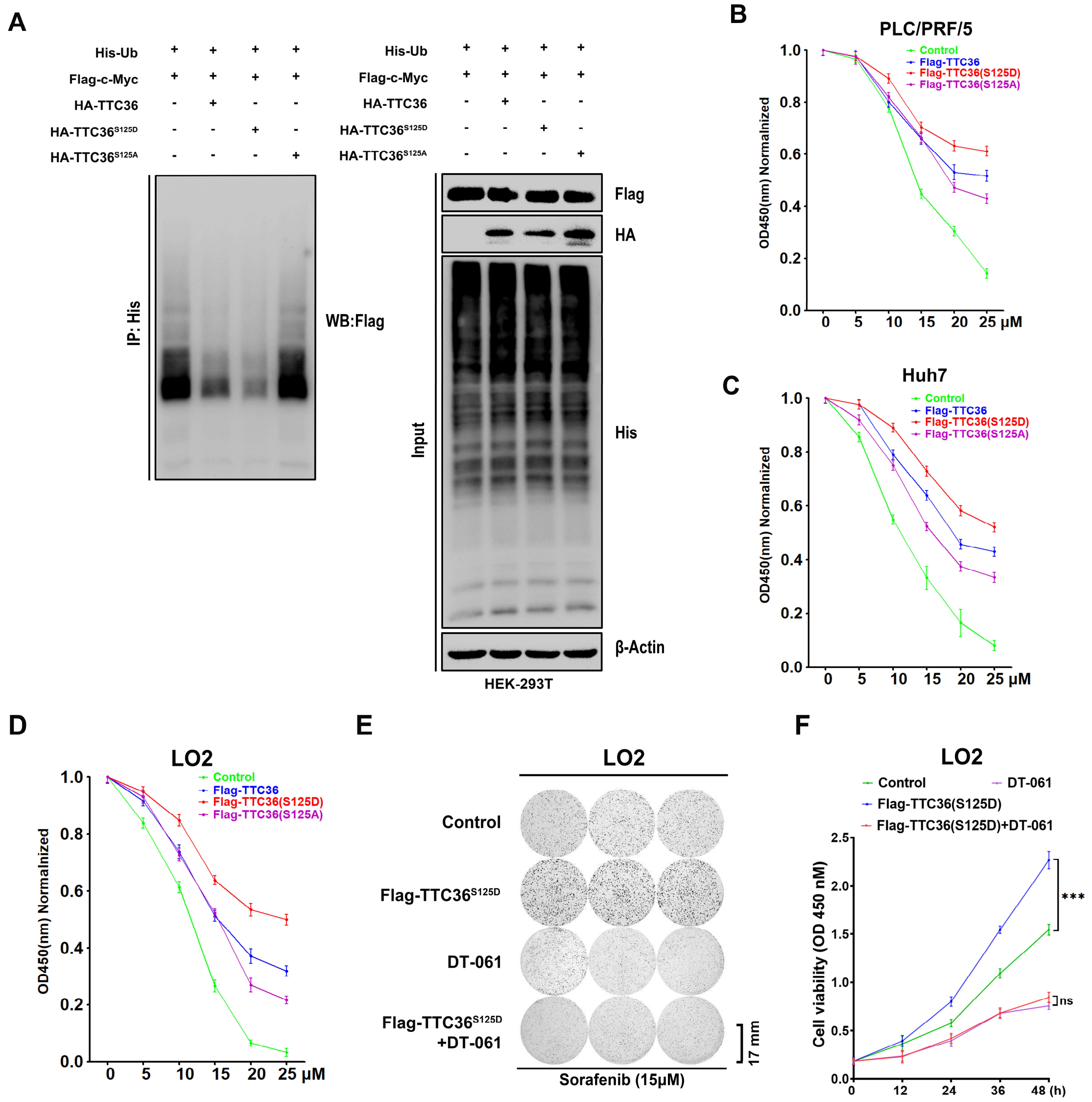

Figure S5. TTC36^{S125D} but not TTC36^{S125A} promotes c-Myc polyubiquitination and HCC proliferation.

(A) HEK-293T cells were transfected with corresponding plasmids, treated with MG-132 (10 μM) for 6 hours, and then harvested with HCl lysis buffer. Immunoprecipitation was conducted using Ni-NTA affinity resin, followed by Western blotting with the indicated antibodies.

(B-D) Assessment of in vitro growth of PLC/PRF5 (B), Huh7 (C) and LO2 (D) cells using Cell Counting Kit-8 under the specified conditions shown in the figure.

(E) Colony formation assay of LO2 cells under the conditions illustrated.

(F) Evaluation of in vitro growth of LO2 cells under the conditions depicted in the figure using the Cell Counting Kit-8.

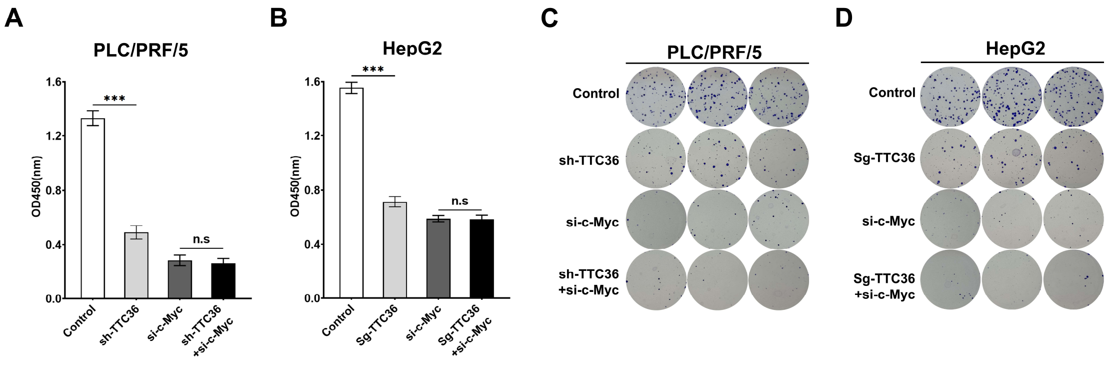

Figure S6. TTC36 promotes cell proliferation and tumor formation in a c-Myc-dependent manner.

(A, B) The effects of sh-TTC36 or Sg-TTC36 inhibition on the in vitro growth of hepatocellular carcinoma cells were evaluated. Potential blocking of this inhibition by si-c-Myc was evaluated using Cell Counting Kit-8.

(C, D) A colony formation assay was used to detect whether si- c-Myc would hinder the growth-promoting effect of sh-TTC36 or Sg-TTC36 inhibition hepatoma cells.

Supplementary Tables

Supplementary Table 1. Chemicals and antibodies used in this study.

Supplementary Table 2. Primers for DNA amplification

Supplementary Table 3. Primers for site-directed mutagenesis

Supplementary Table 4. Primers for gene knockout (sgRNA)

Supplementary Table 5. Primers for gene knockdown (siRNA)

Supplementary Table 6. Primers for Q-PCR.

Supplementary Table 1. Chemicals and antibodies used in this study.

List	Antibodies/chemical	Dilution	Vendors	Catalog no.
Chemical	Cycloheximide	50 μg/ml	Sigma	239764
	MG-132	10 μM	Sigma	474790
	Guanidine hydrochloride	6 mol/L	Sigma	1302134
	5-Azacytidine	3 Mm	MCE	HY-10586
	BIO	5 nM	MCE	HY-10580
	FTY-720	10 μmol/L	MCE	HY-134976
	sorafenib	5-40μmol/L	MCE	HY-10201
	DT-061	10 μmol/l	MCE	HY-112929
Antibody	c-Myc	1:1000	abcam	ab32072
	p-c-Myc^{T58}	1:1000	abcam	ab185655
	p-c-Myc^{S62}	1:1000	abcam	ab185656
	TTC36	1:500	Made in ouself	
	β-Actin	1:1000	Transgen	HC201-01
	Flag	1:1000	Transgen	HT201-01
	GFP	1:1000	Transgen	HT801-01
	His	1:1000	Transgen	HT501-01
	HA	1:1000	Transgen	HT301-01
	FBXW7	1:1000	Abclonal	A5872
	p-GSK3β^{S9}	1:1000	abcam	ab75814
	GSK3β	1:1000	abcam	ab227208
	SET	1:1000	Proteintech	55201-1-AP
	PPP2R1A	1:1000	Proteintech	15882-1-AP
	PPP2CA	1:1000	Proteintech	13482-1-AP
	PPP2CB	1:1000	Proteintech	12554-2-AP

Supplementary Table 2. Primers for DNA amplification

Primer Name	Sequence
H-TTC36-F	ggatccgaattcatggggactccaaatgatcagg
H-TTC36-R	atggtgatggtggtgtcagcggctgtcacgggggc
H-c-Myc-F	taagggatccgaattcctggattttttcgggtagtggaaaacc
H-c-Myc-R	gatggtggtgctcgagttacgcacaagagttccgtagctgt
H-FBXW7-F	taagggatccgaattcatgtcaaaaccgggaaaacctactct
H-FBXW7-R	gatggtggtgctcgagtcacttcatgtccacatcaaagtcca
H-PPP2CA-F	taagggatccgaattcatggacgagaaggtgttcaccaagg
H-PPP2CA-R	gatggtggtgctcgagttacaggaagtagtctggggtacga
H-PPP2CB-F	taagggatccgaattcatggacgacaaggcgttcacc
H-PPP2CB-R	gatggtggtgctcgagttataggaagtagtctggggtgcgc
H-SET-F	taagggatccgaattcatggcccctaaacgccag
H-SET-R	gatggtggtgctcgagttagtcatcttctccttcatcctcc
H-PPP2R1A-F	taagggatccgaattatggtgcggcgggccg
H-PPP2R1A-R	gatggtggtgctcgagtcaggcgagagacagaacagtcag

Supplementary Table 3. Primers for site-directed mutagenesis

Primer Name	Sequence
H-TTC36 S125A-F	gggccgcgccgcccgccaggcctttgtgcagcgcggactcc
H-TTC36 S125D-F	gggccgcgccgcccgccaggactttgtgcagcgcggactcc
H-TTC36 S125A-R	ggagtccgcgctgcacaaaggcctggcgggcggcgcggccc
H-TTC36 S125D-R	ggagtccgcgctgcacaaagtcctggcgggcggcgcggccc

Supplementary Table 4. Primers for gene knockout (sgRNA)

Primer Name	Sequence
sgRNA-TTC36-F#1	caccgcttgagggaaaacttcatct
sgRNA-TTC36-R#1	aaacagatgaagttttccctcaagc
sgRNA-TTC36-F#2	caccgagttttccctcaagcacagc
sgRNA-TTC36-R#2	aaacgctgtgcttgagggaaaactc
sgRNA-TTC36-F#3	caccgactgttccagctgtgcttga
sgRNA-TTC36-R#3	aaactcaagcacagctggaacagtc

Supplementary Table 5. Primers for gene knockdown (siRNA)

Primer Name	Sequence
si-PPP2R1A#1	uuccacuagcuucuucagg
	ccugaagaagcuaguggaa
si-PPP2R1A#2	uucacagaacucuuugacc
	ggucaaagaguucugugaa
si-PPP2R1A#3	uucacagcacuggacacuc
	gaguguccagugcugugaa
si-GSK3β#1	gcuauacagacacuaaagu
	acuuuagugucuguauagc
si-GSK3β#2	gcuagaucacuguaacaua
	uauguuacagugaucuagc
si-GSK3β#3	guuacuaggacaaccaaua
	uauugguuguccuaguaac
si-c-Myc#1	ggaacuaugaccucgacuacg
	uagucgaggucauaguuccug
si-c-Myc#2	gcgaggauaucuggaagaaau
	uucuuccagauauccucgcug

Supplementary Table 6. Primers for Q-PCR.

Primer Name	Sequence
TTC36	cgagaagaagatgaagttttccct
	gcacggttgttgtaggctga
c-Myc	agcgactctgaggaggaaca
	ccctcttggcagcaggatag
