# Supplementary material for: TTC36 promotes proliferation and drug resistance in hepatocellular carcinoma cells by inhibiting c-Myc degradation
Source: Cell Death Dis. 2025 Apr 24;16(1):332. doi: 10.1038/s41419-025-07663-4 (PMC12022016; doi:10.1038/s41419-025-07663-4)

Figure 1

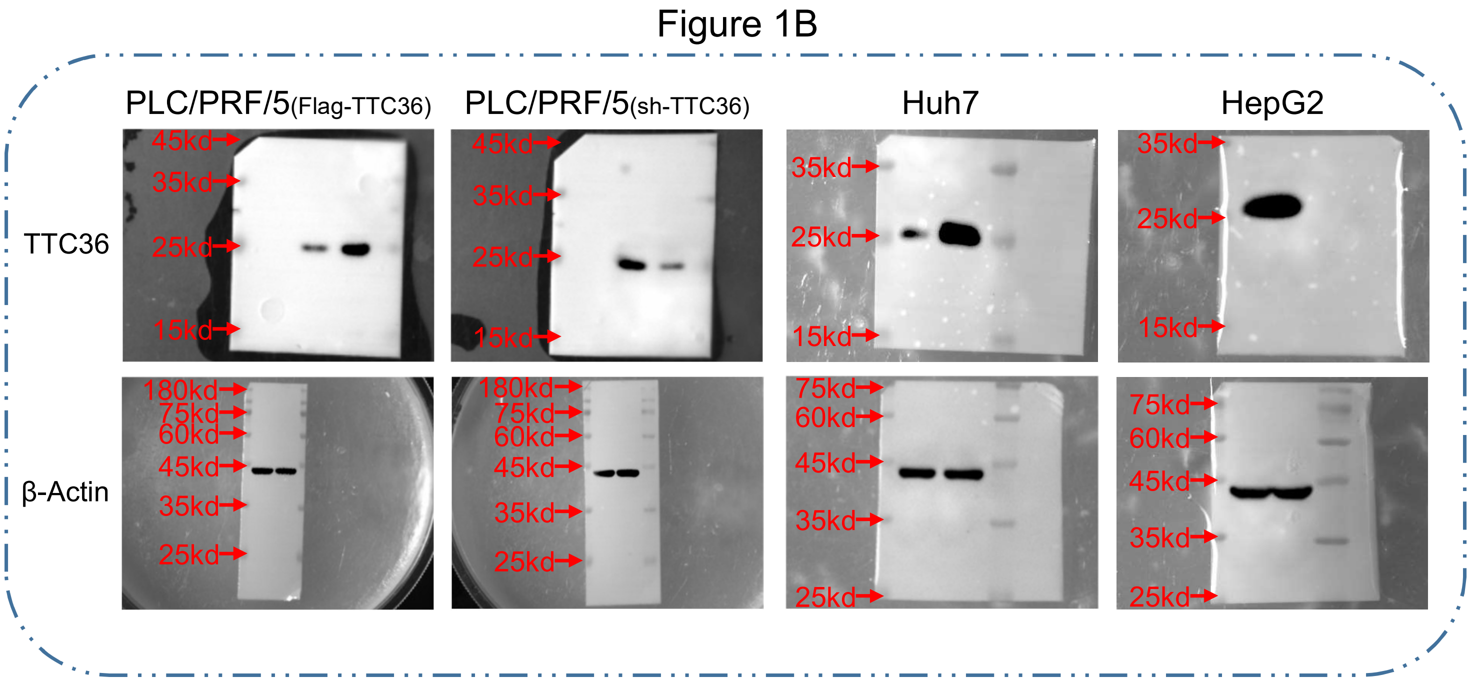
Figure 1B
PLC/PRF/5(Flag-TTC36)
PLC/PRF/5(sh-TTC36)
Huh7
HepG2
TTC36
β-Actin
45kd
35kd
25kd
15kd
180kd
75kd
60kd
45kd
35kd
25kd

Figure 2

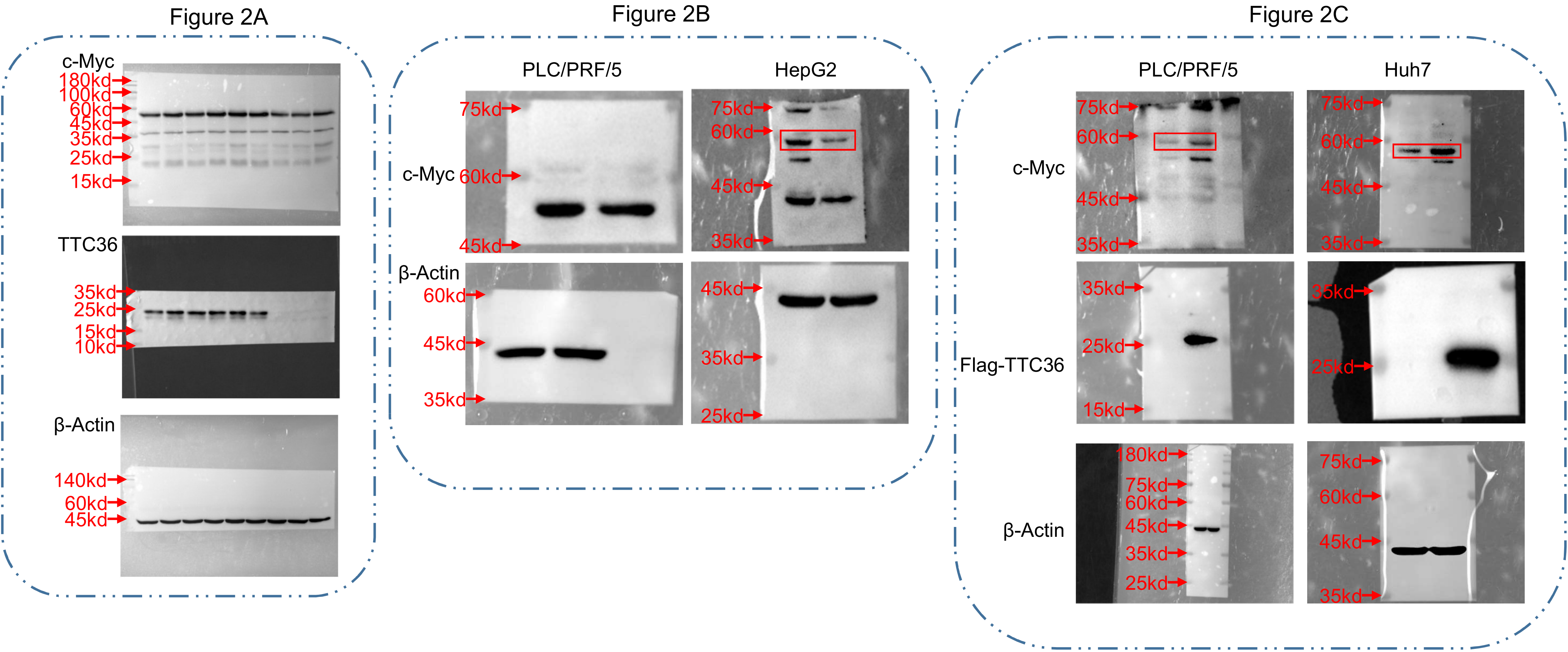

Figure 2A
c-Myc
180kd
100kd
60kd
45kd
35kd
25kd
15kd
TTC36
35kd
25kd
15kd
10kd
β-Actin
140kd
60kd
45kd
Figure 2B
PLC/PRF/5
HepG2
c-Myc
75kd
60kd
45kd
35kd
β-Actin
60kd
45kd
35kd
25kd
Figure 2C
PLC/PRF/5
Huh7
c-Myc
75kd
60kd
45kd
35kd
Flag-TTC36
35kd
25kd
15kd
β-Actin
180kd
75kd
60kd
45kd
35kd
25kd

Figure 2

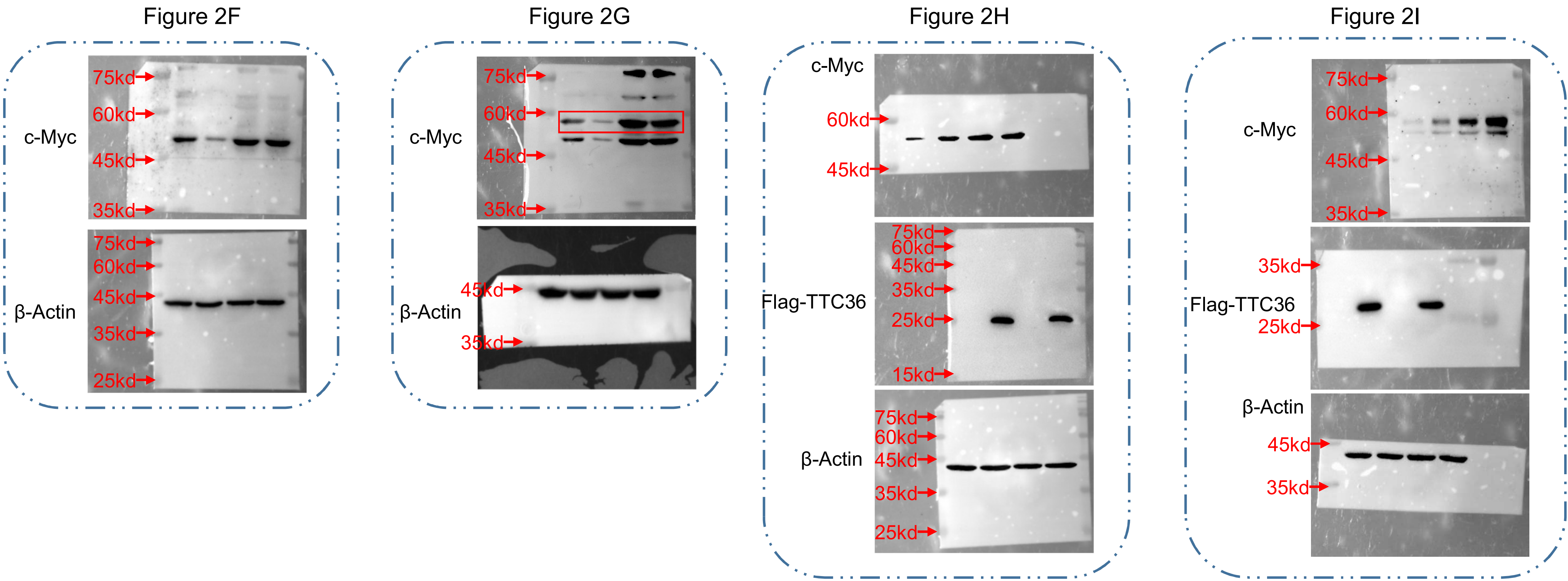

Figure 2F
c-Myc
75kd
60kd
45kd
35kd
β-Actin
75kd
60kd
45kd
35kd
25kd
Figure 2G
c-Myc
75kd
60kd
45kd
35kd
β-Actin
45kd
35kd
Figure 2H
c-Myc
60kd
45kd
Flag-TTC36
75kd
60kd
45kd
35kd
25kd
15kd
β-Actin
75kd
60kd
45kd
35kd
25kd
Figure 2I
c-Myc
75kd
60kd
45kd
35kd
Flag-TTC36
35kd
25kd
β-Actin
45kd
35kd

Figure 2

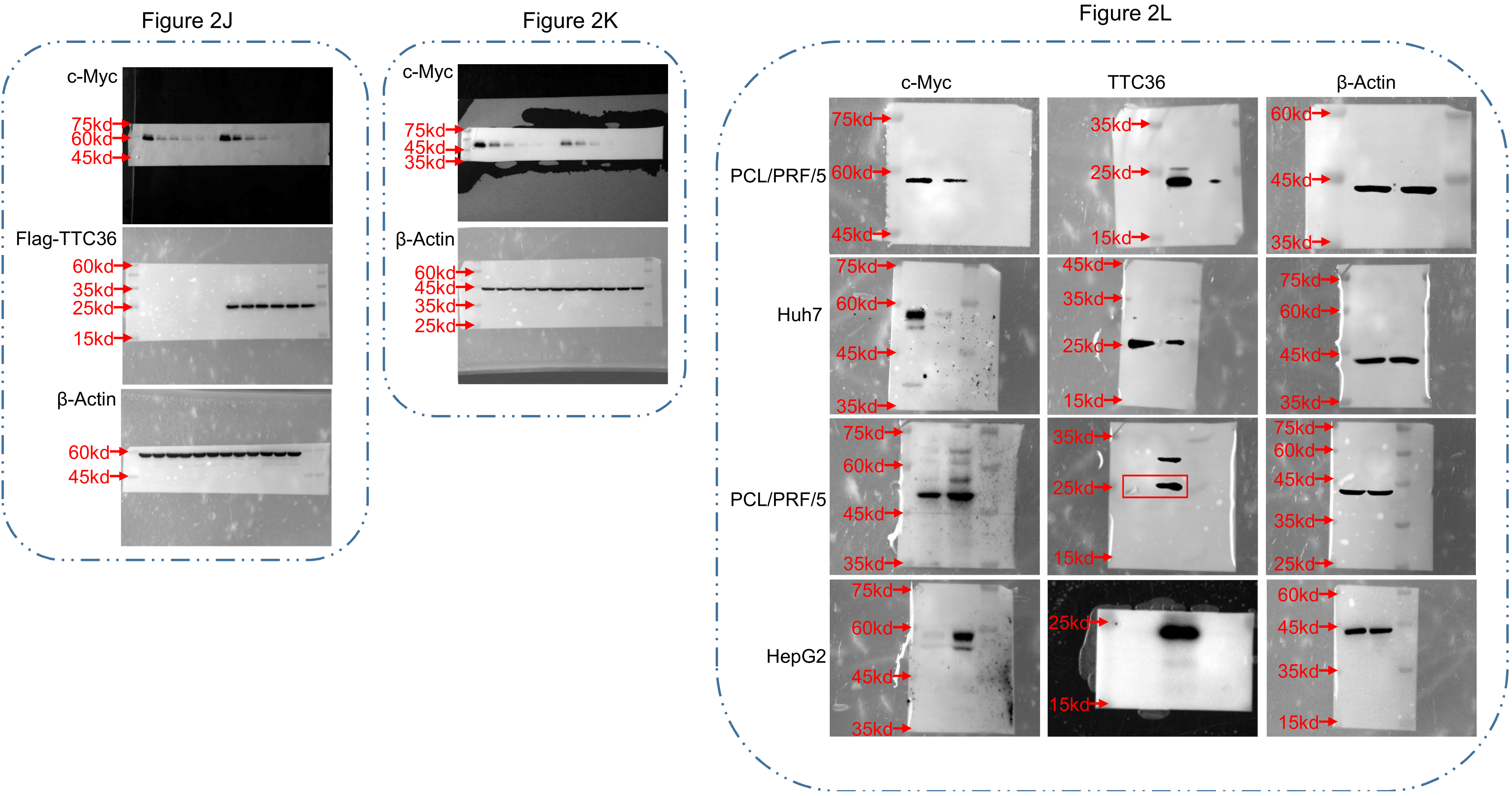

Figure 2J
c-Myc
75kd
60kd
45kd
Flag-TTC36
60kd
35kd
25kd
15kd
β-Actin
60kd
45kd
Figure 2K
c-Myc
75kd
45kd
35kd
β-Actin
60kd
45kd
35kd
25kd
Figure 2L
c-Myc
TTC36
β-Actin
PCL/PRF/5
Huh7
PCL/PRF/5
HepG2
75kd
60kd
45kd
35kd
25kd
15kd

Figure 2

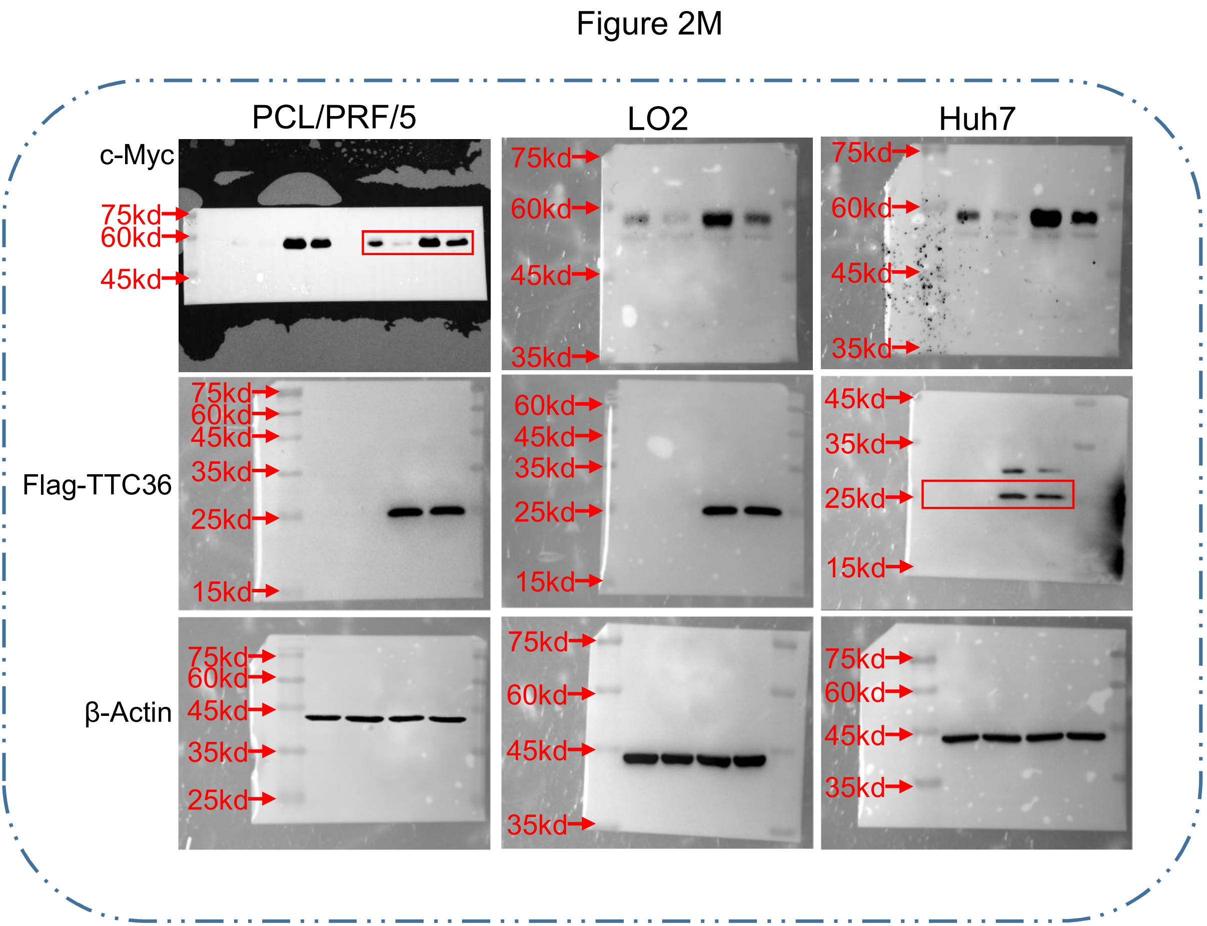

Figure 2M
PCL/PRF/5
LO2
Huh7
c-Myc
Flag-TTC36
β-Actin
75kd
60kd
45kd
35kd
25kd
15kd

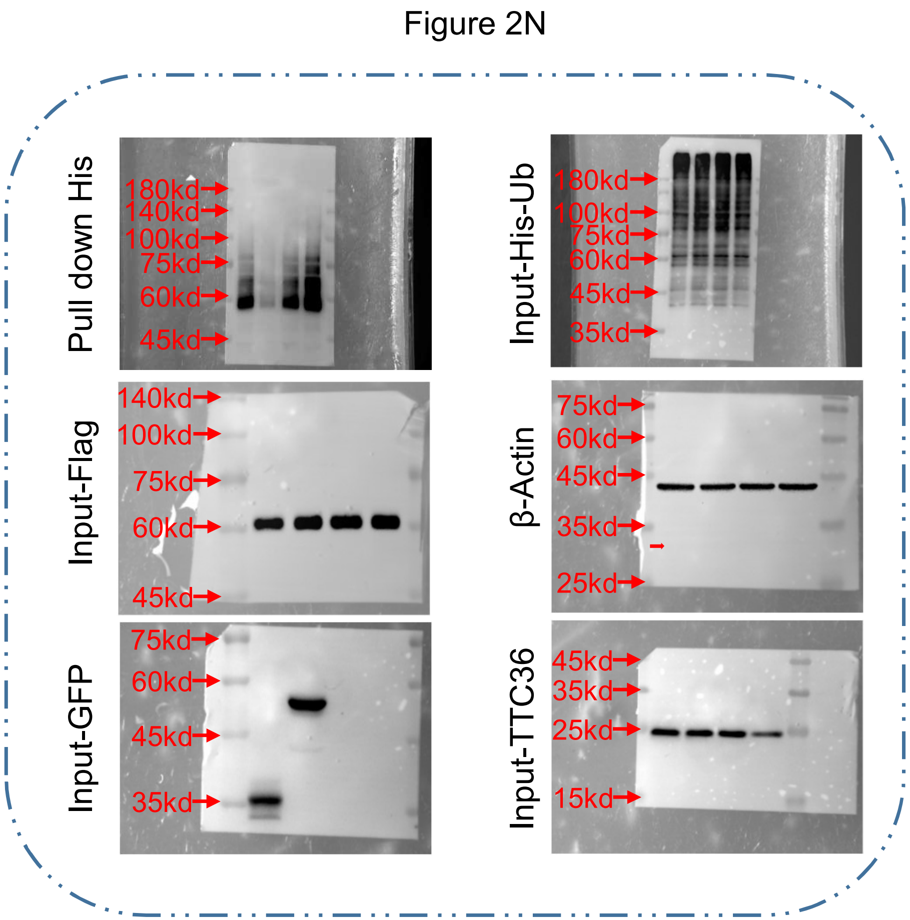

Figure 2N
Pull down His
Input-His-Ub
Input-Flag
β-Actin
Input-GFP
Input-TTC36
180kd
140kd
100kd
75kd
60kd
45kd
35kd
25kd
15kd

Figure 3

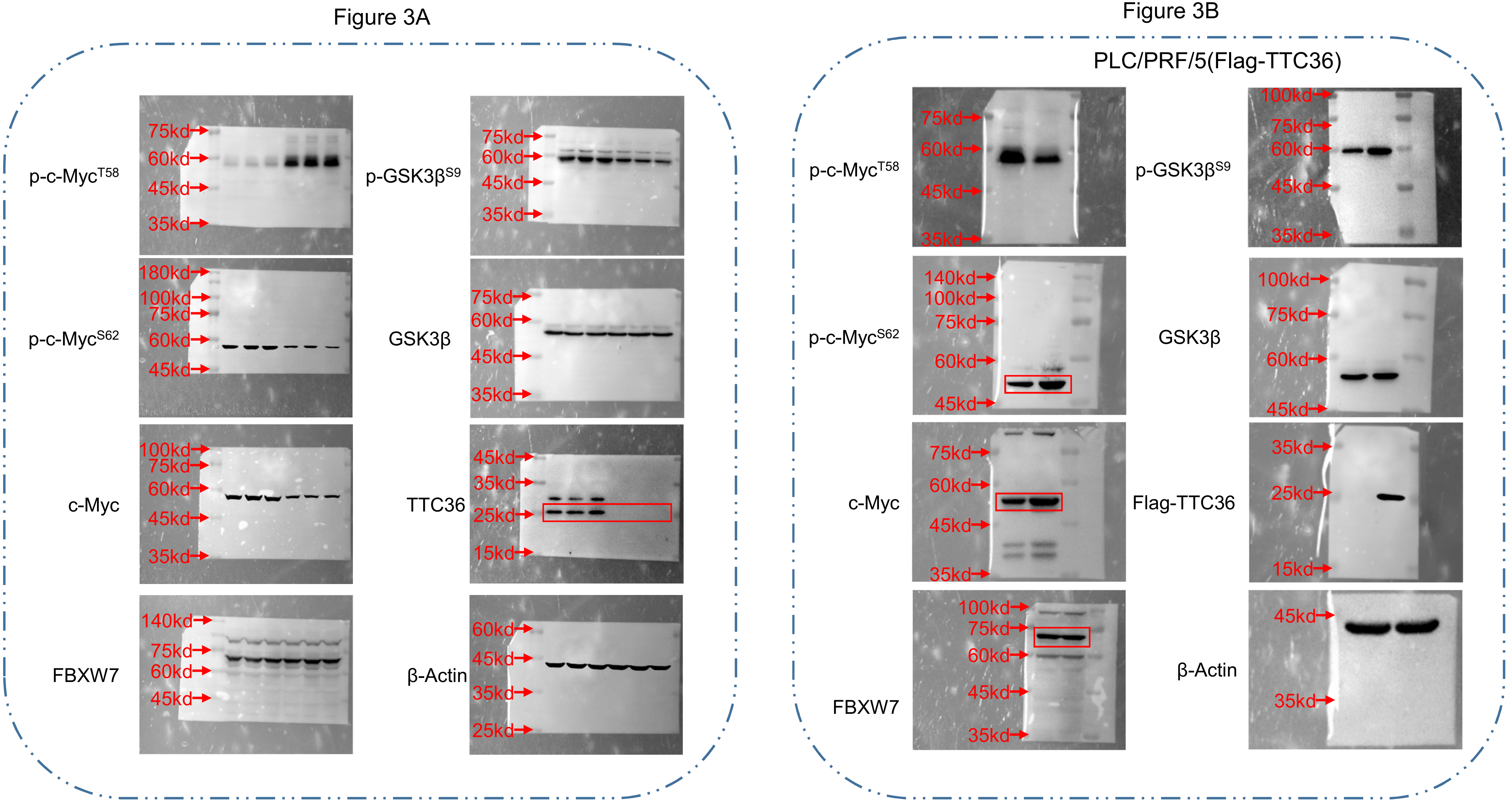
Figure 3A
p-c-Myc^{T58}
p-GSK3β^{S9}
p-c-Myc^{S62}
GSK3β
c-Myc
TTC36
FBXW7
β-Actin
Figure 3B
PLC/PRF/5(Flag-TTC36)
p-c-Myc^{T58}
p-GSK3β^{S9}
p-c-Myc^{S62}
GSK3β
c-Myc
Flag-TTC36
FBXW7
β-Actin

Figure 3

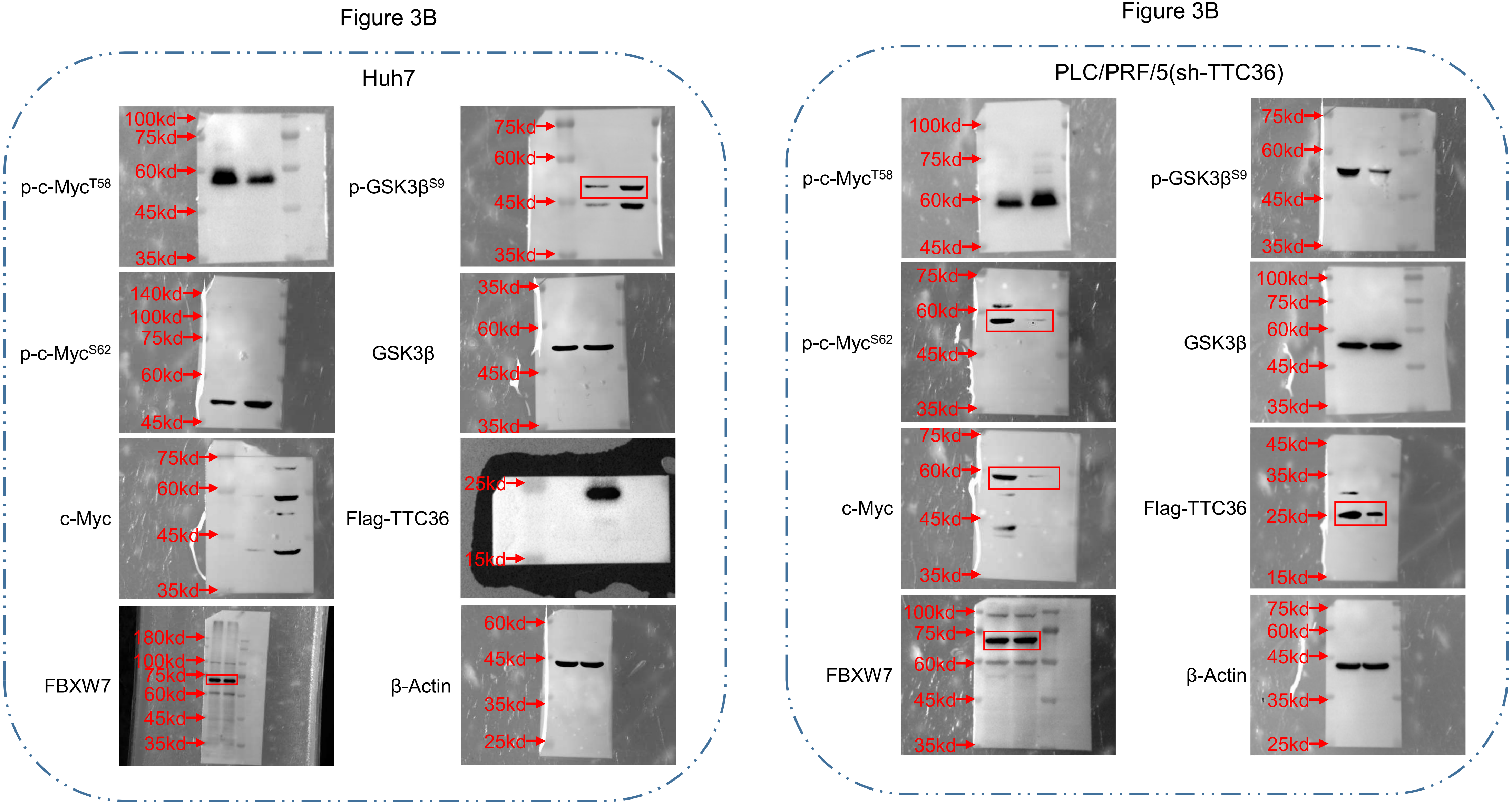

Figure 3B
Huh7
p-c-MycT58
p-GSK3βS9
p-c-MycS62
GSK3β
c-Myc
Flag-TTC36
FBXW7
β-Actin
180kd
140kd
100kd
75kd
60kd
45kd
35kd
25kd
15kd
Figure 3B
PLC/PRF/5(sh-TTC36)
p-c-MycT58
p-GSK3βS9
p-c-MycS62
GSK3β
c-Myc
Flag-TTC36
FBXW7
β-Actin

Figure 3

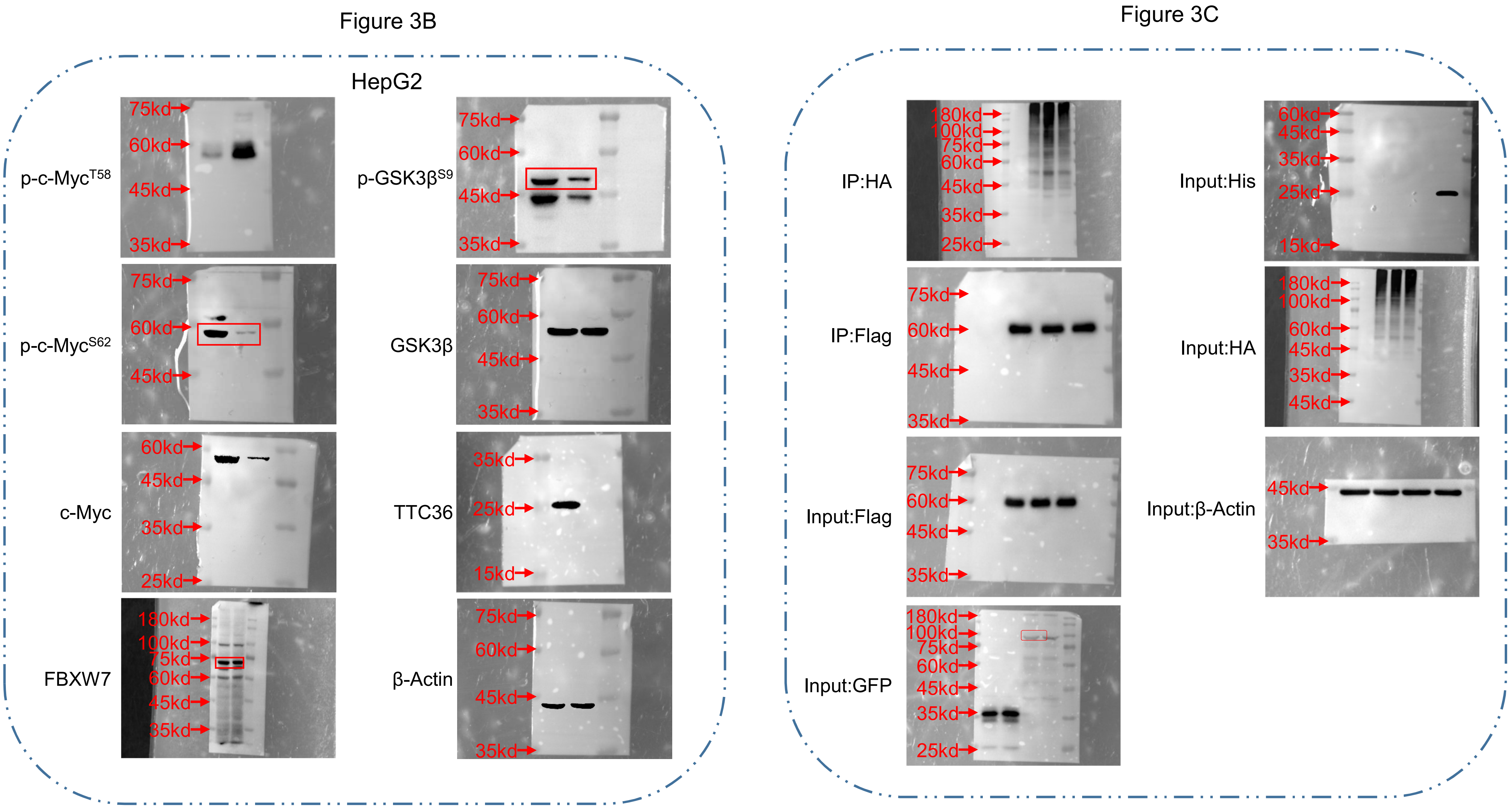
Figure 3B
HepG2
p-c-Myc^{T58}
75kd
60kd
45kd
35kd
p-GSK3β^{S9}
p-c-Myc^{S62}
GSK3β
c-Myc
25kd
TTC36
15kd
FBXW7
180kd
100kd
β-Actin
Figure 3C
IP:HA
Input:His
IP:Flag
Input:HA
Input:Flag
Input:β-Actin
Input:GFP

Figure 3

Figure 3D

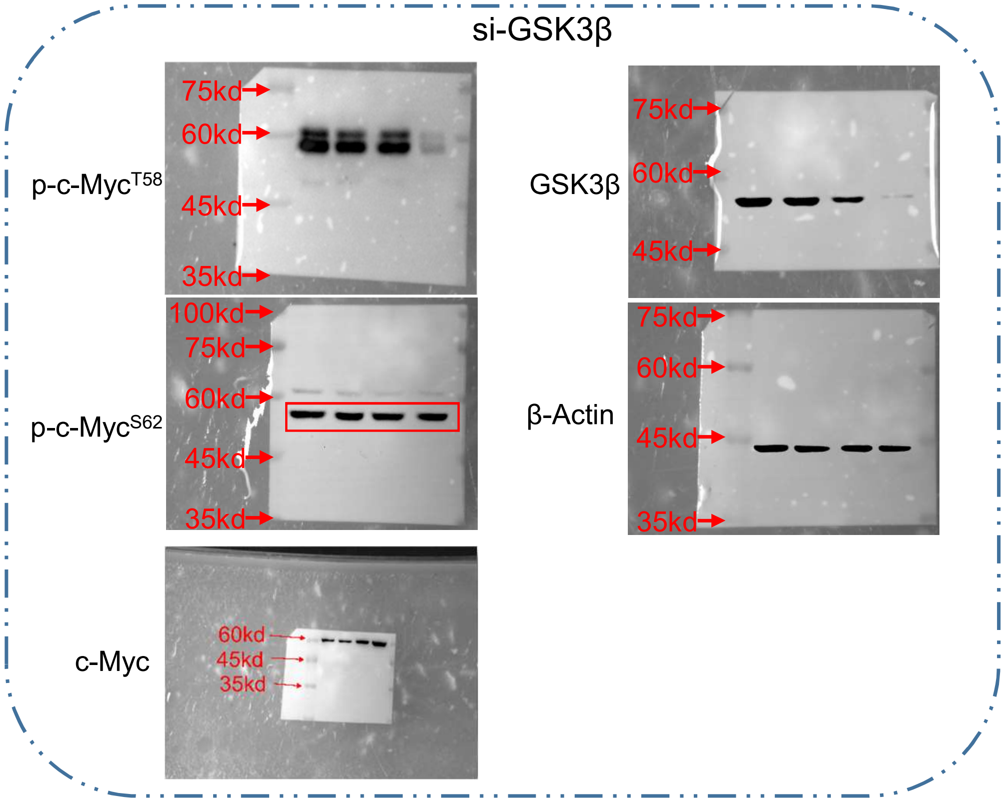

Figure 3D

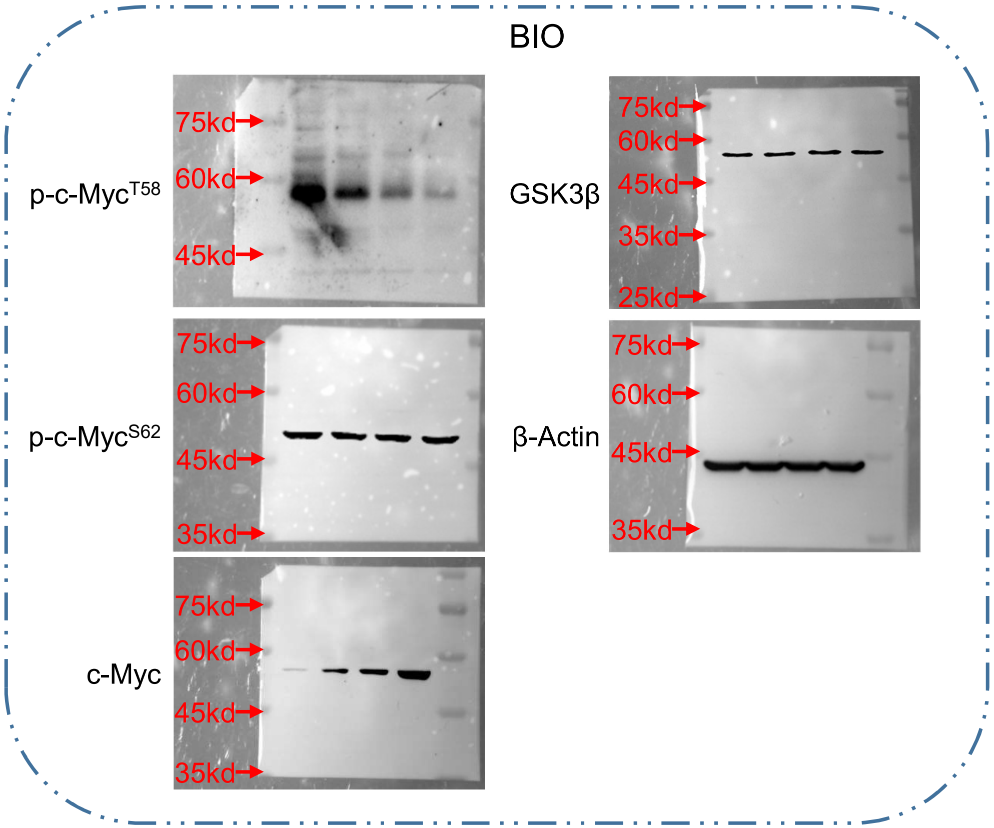

Figure 3

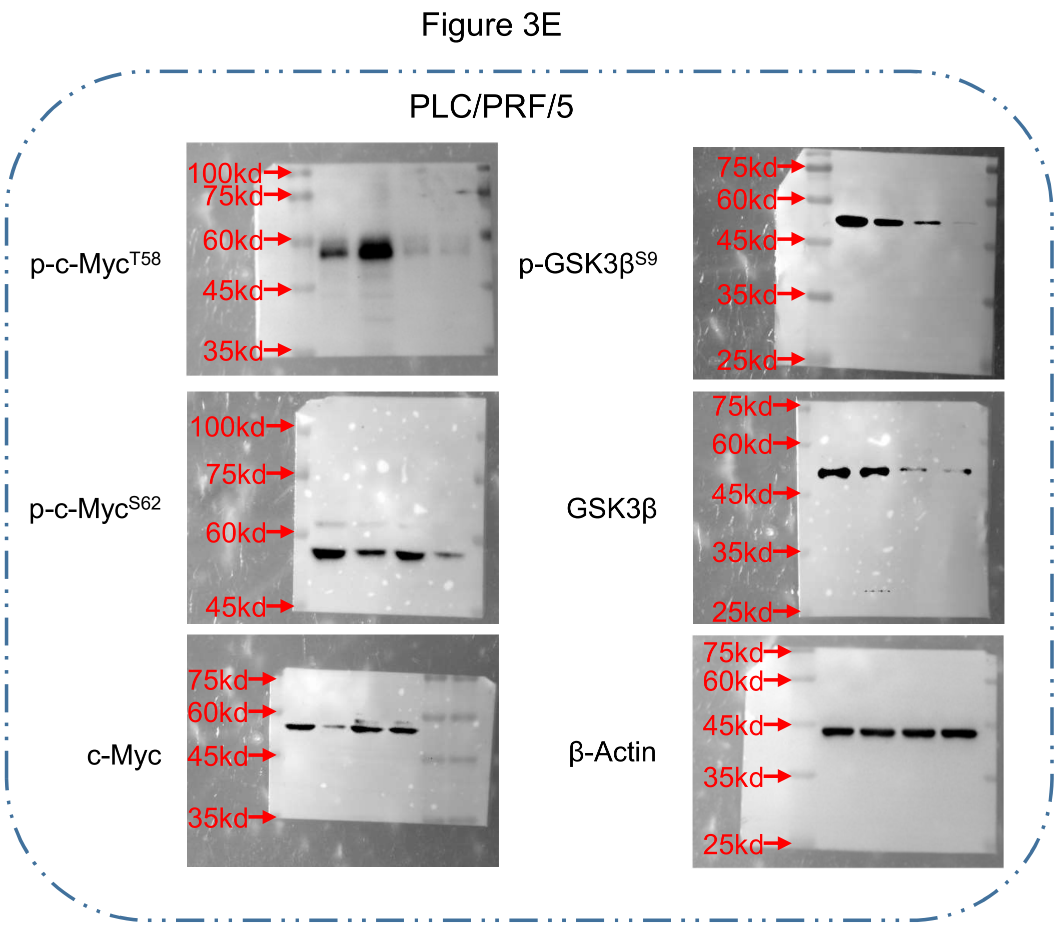
Figure 3E
PLC/PRF/5
p-c-MycT58
p-GSK3βS9
p-c-MycS62
GSK3β
c-Myc
β-Actin
100kd
75kd
60kd
45kd
35kd
25kd

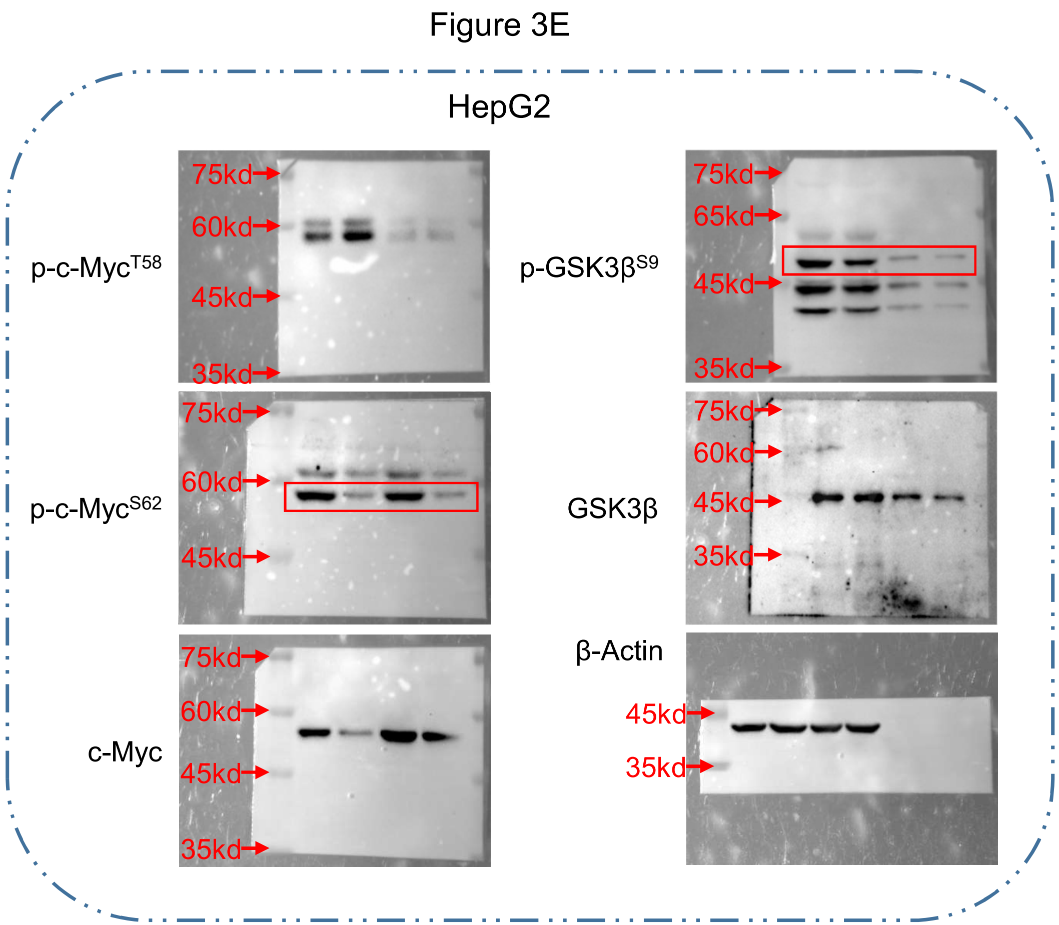
Figure 3E
HepG2
p-c-MycT58
p-GSK3βS9
p-c-MycS62
GSK3β
c-Myc
β-Actin
75kd
65kd
60kd
45kd
35kd

Figure 3

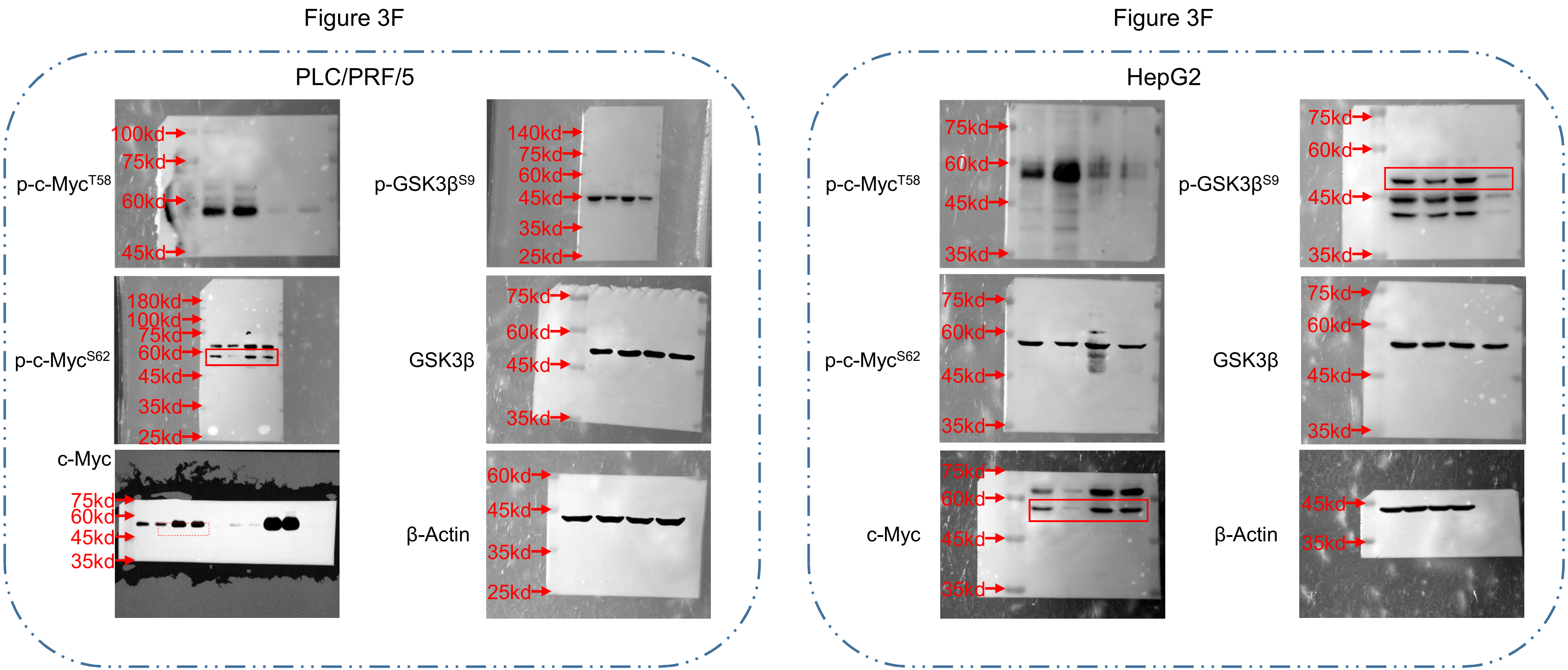
Figure 3F
Figure 3F
PLC/PRF/5
HepG2
p-c-MycT58
p-GSK3βS9
p-c-MycS62
GSK3β
c-Myc
β-Actin
100kd
75kd
60kd
45kd
140kd
35kd
25kd
180kd

Figure 3

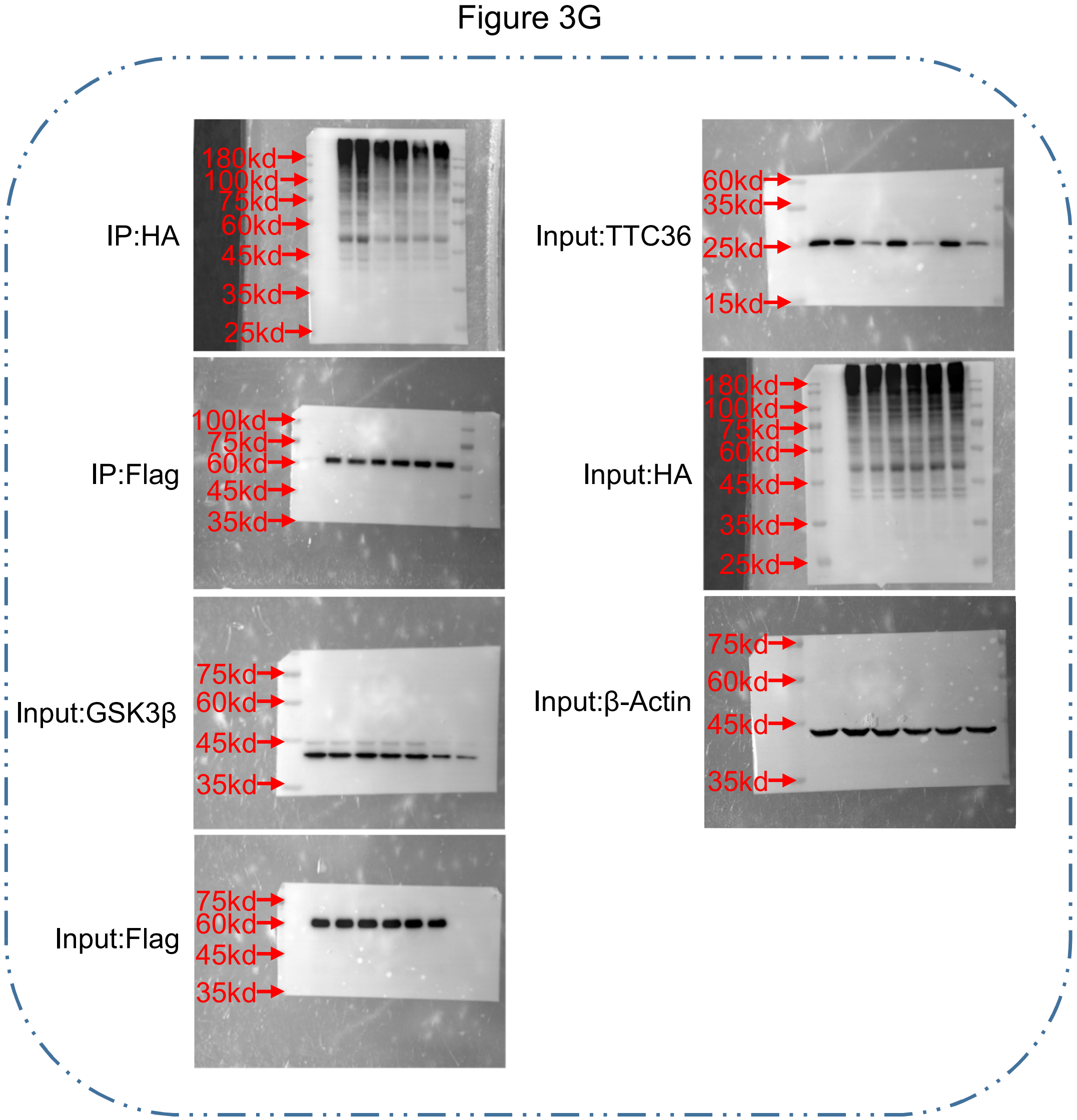

Figure 3G
IP:HA
180kd
100kd
75kd
60kd
45kd
35kd
25kd
Input:TTC36
60kd
35kd
25kd
15kd
IP:Flag
100kd
75kd
60kd
45kd
35kd
Input:HA
180kd
100kd
75kd
60kd
45kd
35kd
25kd
Input:GSK3β
75kd
60kd
45kd
35kd
Input:β-Actin
75kd
60kd
45kd
35kd
Input:Flag
75kd
60kd
45kd
35kd

Figure 5

Figure 5A

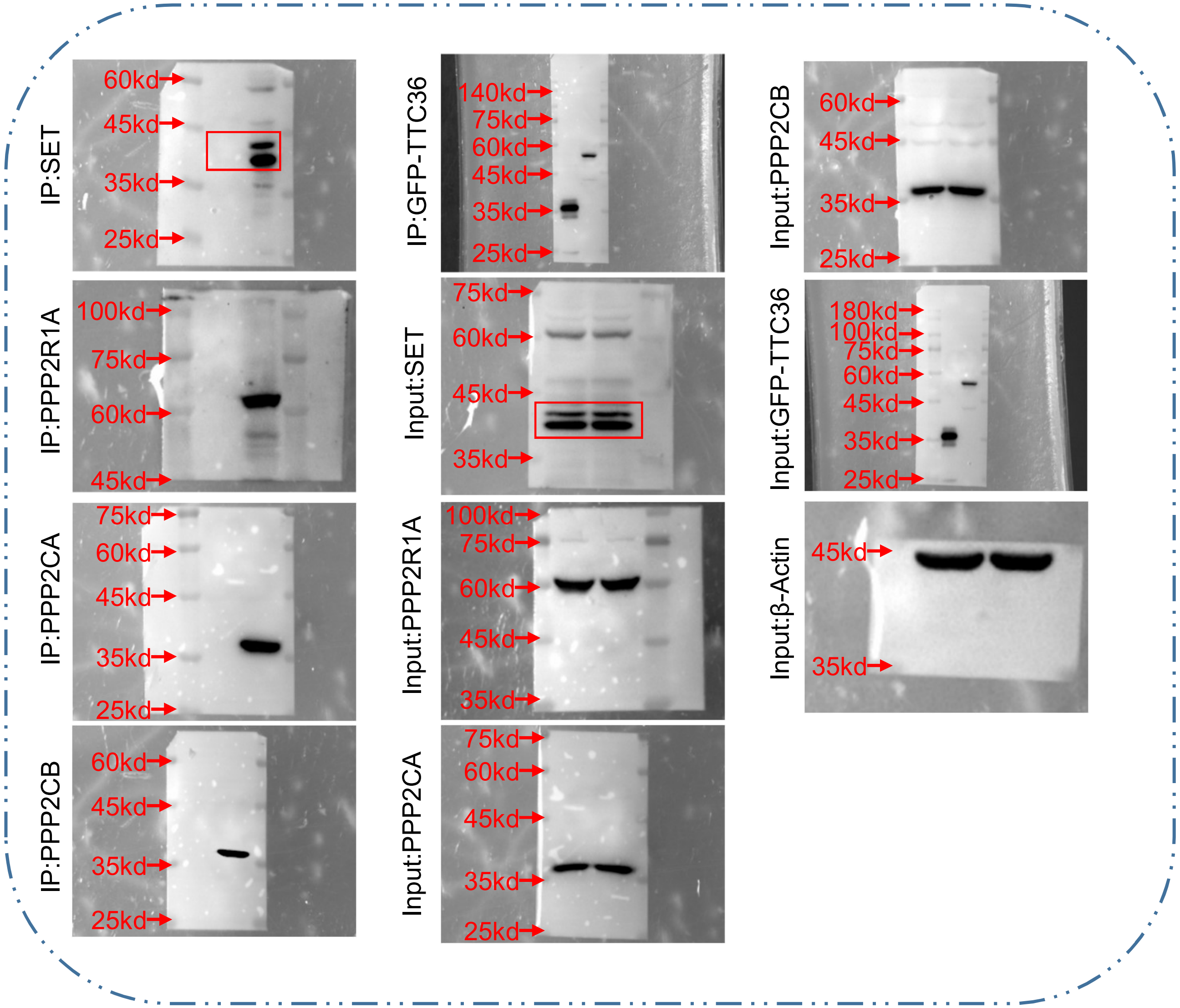

Figure 5B

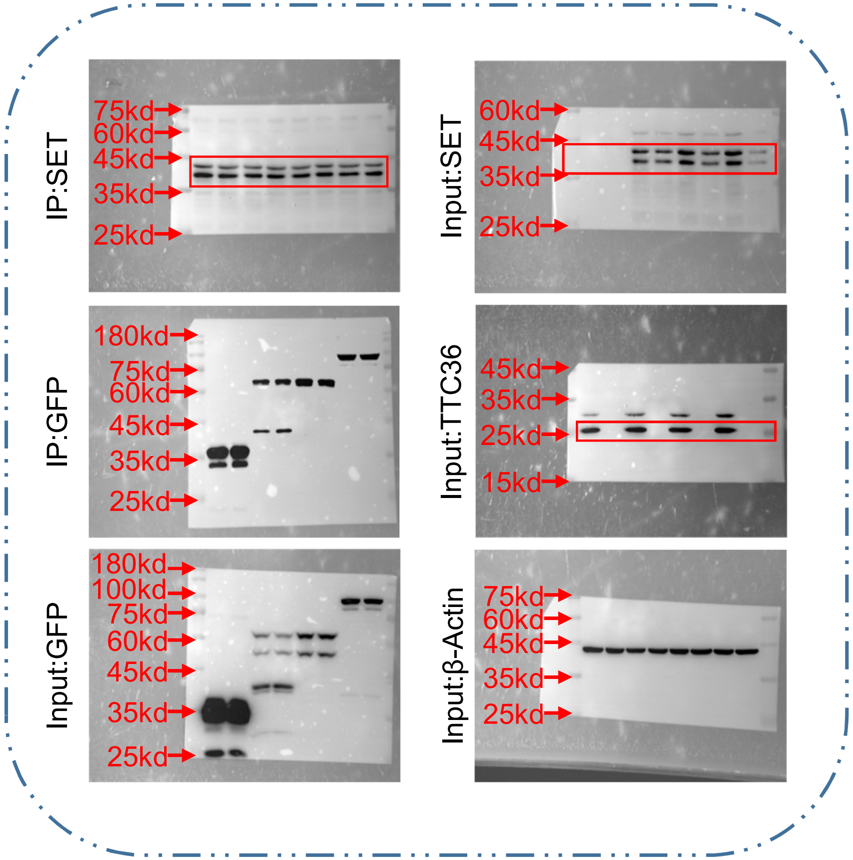

Figure 5

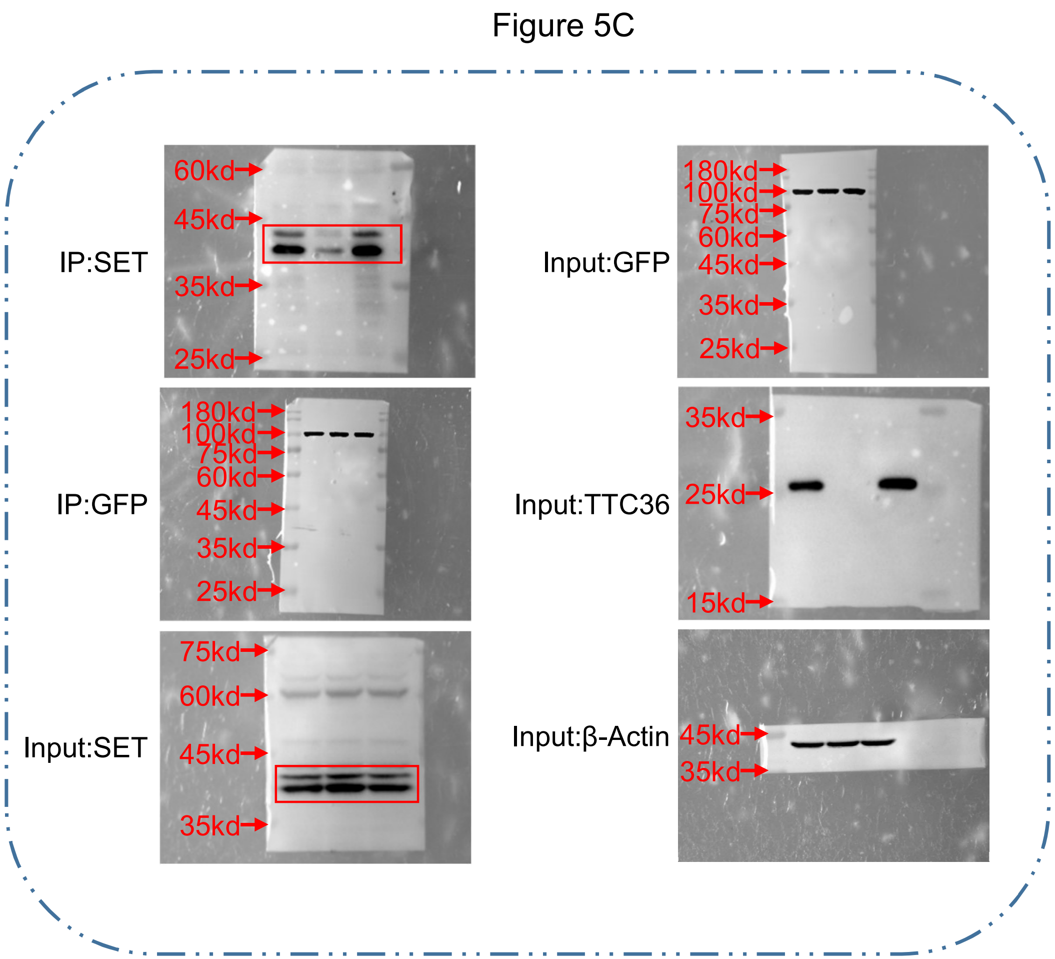
Figure 5C
IP:SET
60kd
45kd
35kd
25kd
Input:GFP
180kd
100kd
75kd
60kd
45kd
35kd
25kd
IP:GFP
180kd
100kd
75kd
60kd
45kd
35kd
25kd
Input:TTC36
35kd
25kd
15kd
Input:SET
75kd
60kd
45kd
35kd
Input:β-Actin
45kd
35kd

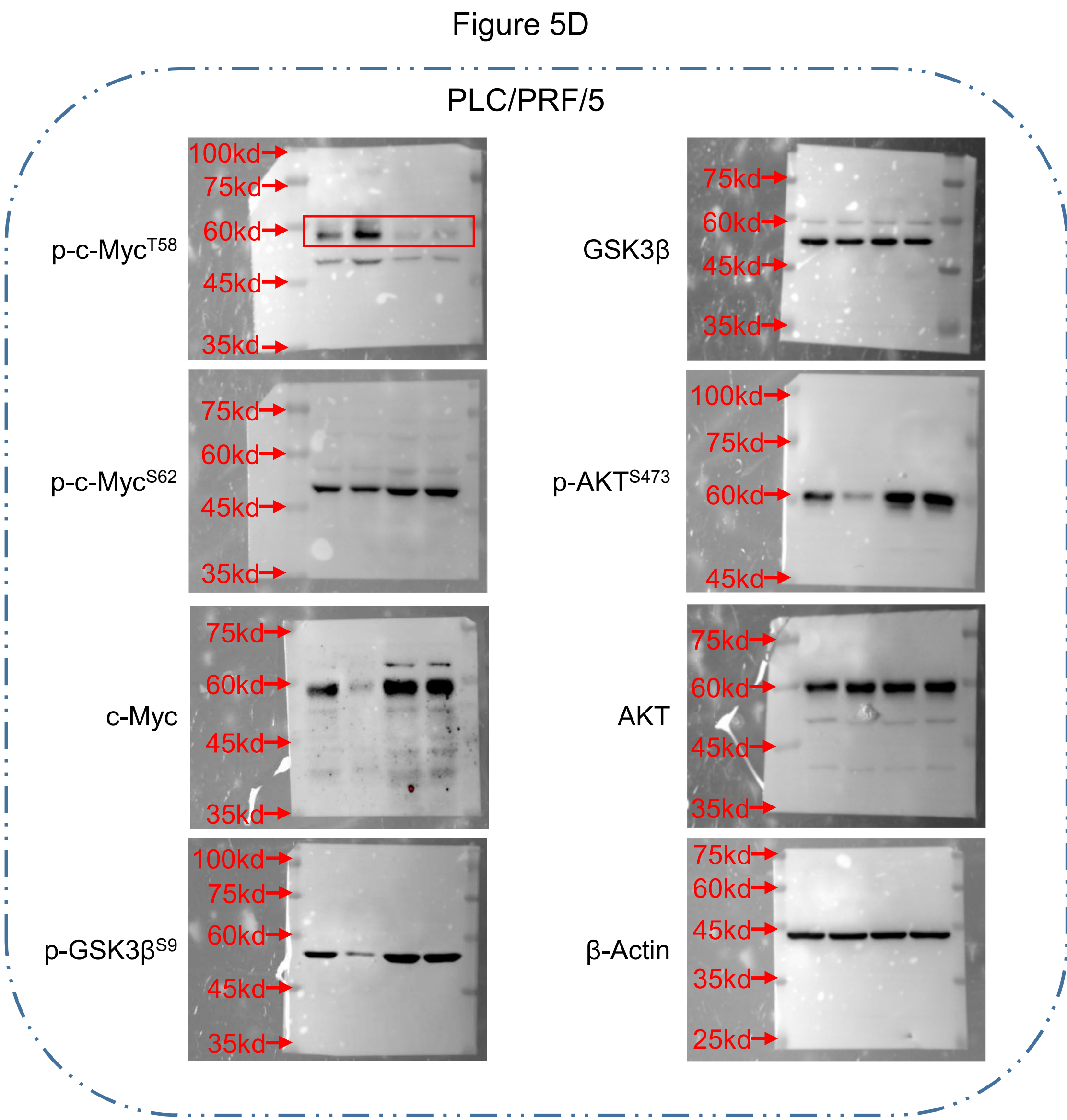
Figure 5D
PLC/PRF/5
p-c-MycT58
100kd
75kd
60kd
45kd
35kd
GSK3β
75kd
60kd
45kd
35kd
p-c-MycS62
75kd
60kd
45kd
35kd
p-AKTS473
100kd
75kd
60kd
45kd
c-Myc
75kd
60kd
45kd
35kd
AKT
75kd
60kd
45kd
35kd
p-GSK3βS9
100kd
75kd
60kd
45kd
35kd
β-Actin
75kd
60kd
45kd
35kd
25kd

Figure 5

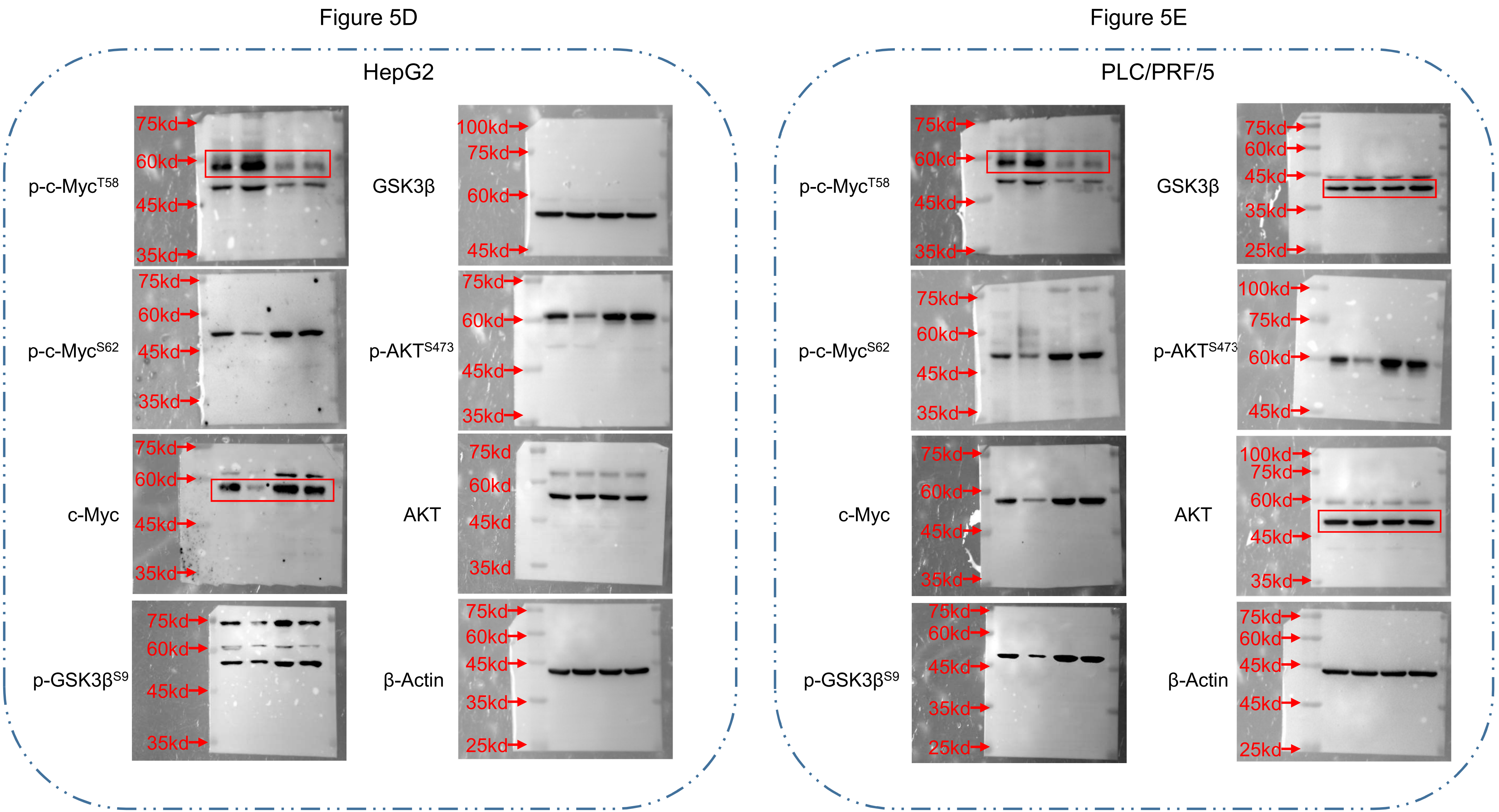
Figure 5D
HepG2
p-c-MycT58
GSK3β
p-c-MycS62
p-AKTS473
c-Myc
AKT
p-GSK3βS9
β-Actin
Figure 5E
PLC/PRF/5
p-c-MycT58
GSK3β
p-c-MycS62
p-AKTS473
c-Myc
AKT
p-GSK3βS9
β-Actin
100kd
75kd
60kd
45kd
35kd
25kd

Figure 5

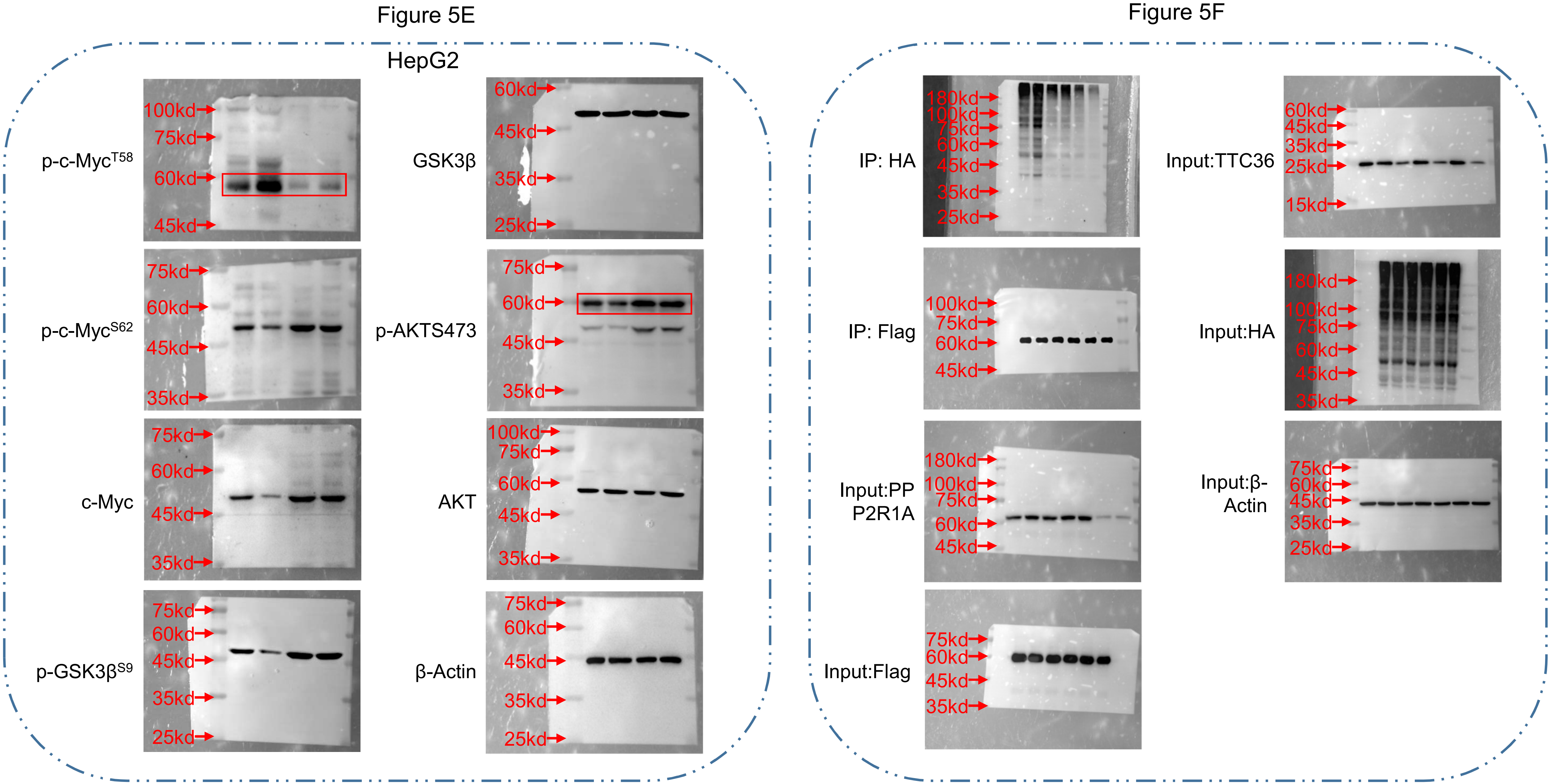

Figure 5E
HepG2
p-c-MycT58
100kd
75kd
60kd
45kd
GSK3β
60kd
45kd
35kd
25kd
p-c-MycS62
75kd
60kd
45kd
35kd
p-AKTS473
75kd
60kd
45kd
35kd
c-Myc
75kd
60kd
45kd
35kd
AKT
100kd
75kd
60kd
45kd
35kd
p-GSK3βS9
75kd
60kd
45kd
35kd
25kd
β-Actin
75kd
60kd
45kd
35kd
25kd
Figure 5F
IP: HA
180kd
100kd
75kd
60kd
45kd
35kd
25kd
Input:TTC36
60kd
45kd
35kd
25kd
15kd
IP: Flag
100kd
75kd
60kd
45kd
Input:HA
180kd
100kd
75kd
60kd
45kd
35kd
Input:PP P2R1A
180kd
100kd
75kd
60kd
45kd
Input:β-Actin
75kd
60kd
45kd
35kd
25kd
Input:Flag
75kd
60kd
45kd
35kd

Figure 6

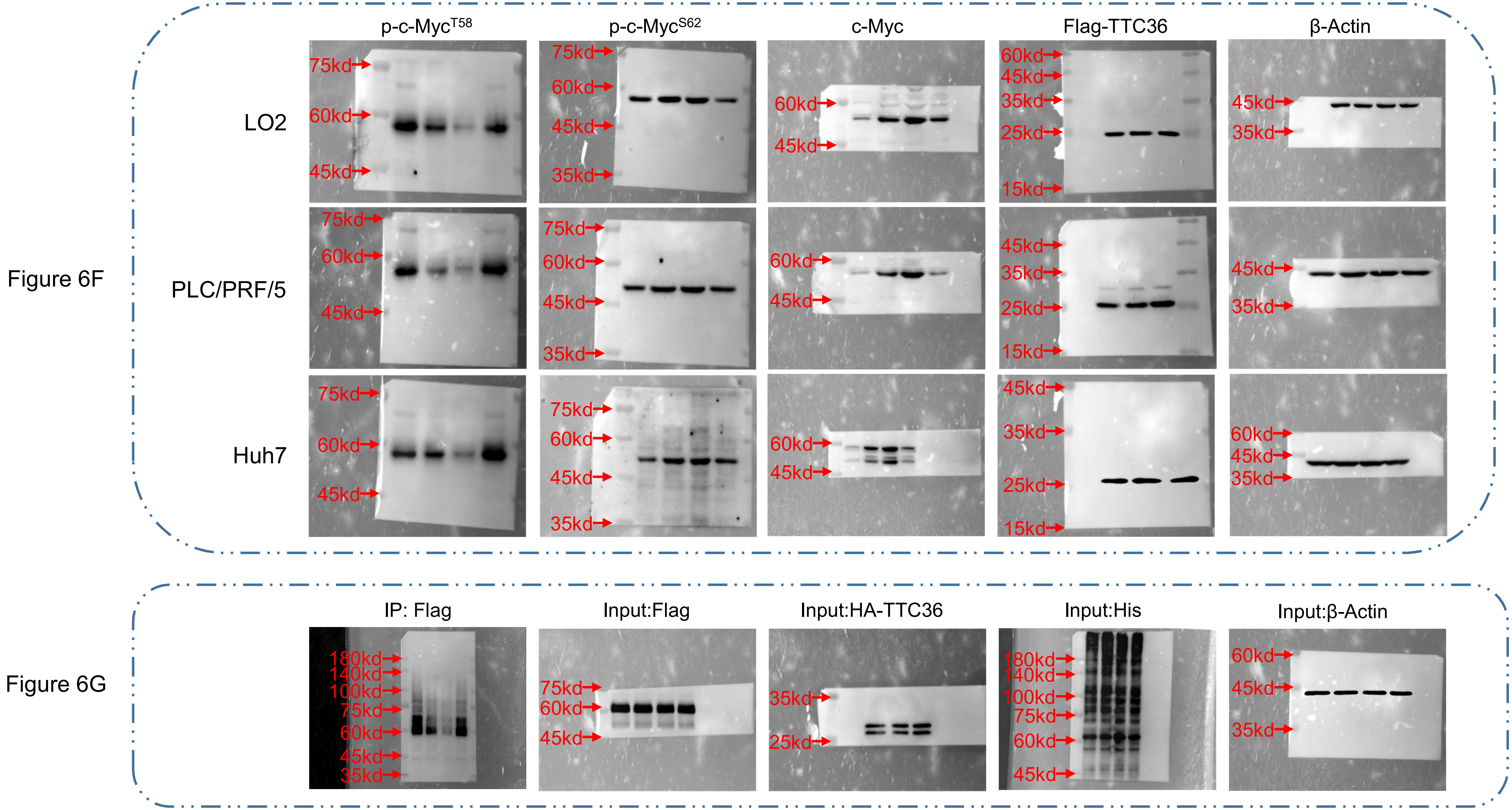

Figure 6F
LO2
PLC/PRF/5
Huh7
p-c-MycT58
p-c-MycS62
c-Myc
Flag-TTC36
β-Actin
Figure 6G
IP: Flag
Input:Flag
Input:HA-TTC36
Input:His
Input:β-Actin
180kd
140kd
100kd
75kd
60kd
45kd
35kd
25kd
15kd

Figure 7

Figure 7

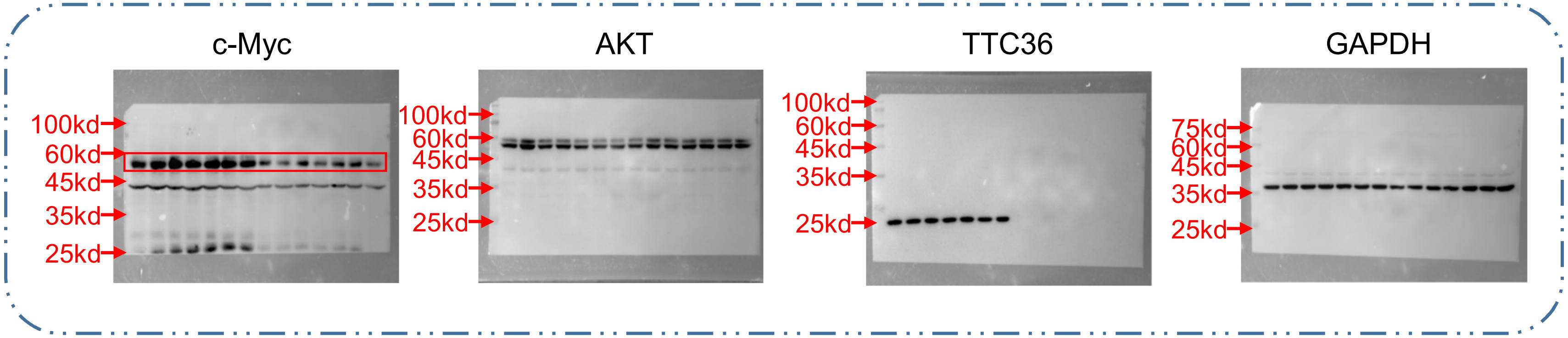

Figure S2

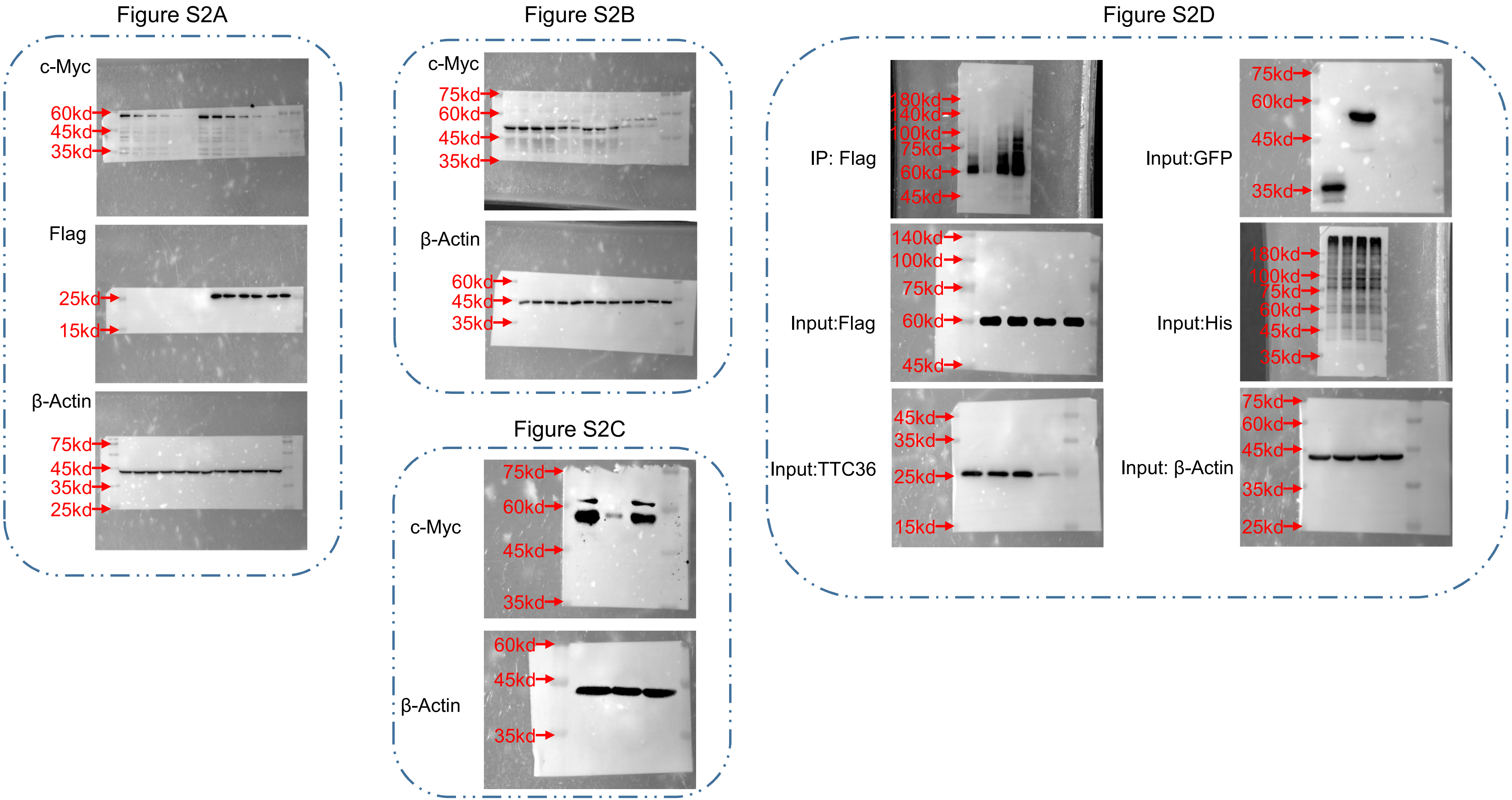
Figure S2A
c-Myc
60kd
45kd
35kd
Flag
25kd
15kd
β-Actin
75kd
45kd
35kd
25kd
Figure S2B
c-Myc
75kd
60kd
45kd
35kd
β-Actin
60kd
45kd
35kd
Figure S2C
c-Myc
75kd
60kd
45kd
35kd
β-Actin
60kd
45kd
35kd
Figure S2D
IP: Flag
180kd
140kd
100kd
75kd
60kd
45kd
Input:GFP
75kd
60kd
45kd
35kd
Input:Flag
140kd
100kd
75kd
60kd
45kd
Input:His
180kd
100kd
75kd
60kd
45kd
35kd
Input:TTC36
45kd
35kd
25kd
15kd
Input: β-Actin
75kd
60kd
45kd
35kd
25kd

Figure S3

Figure S3A

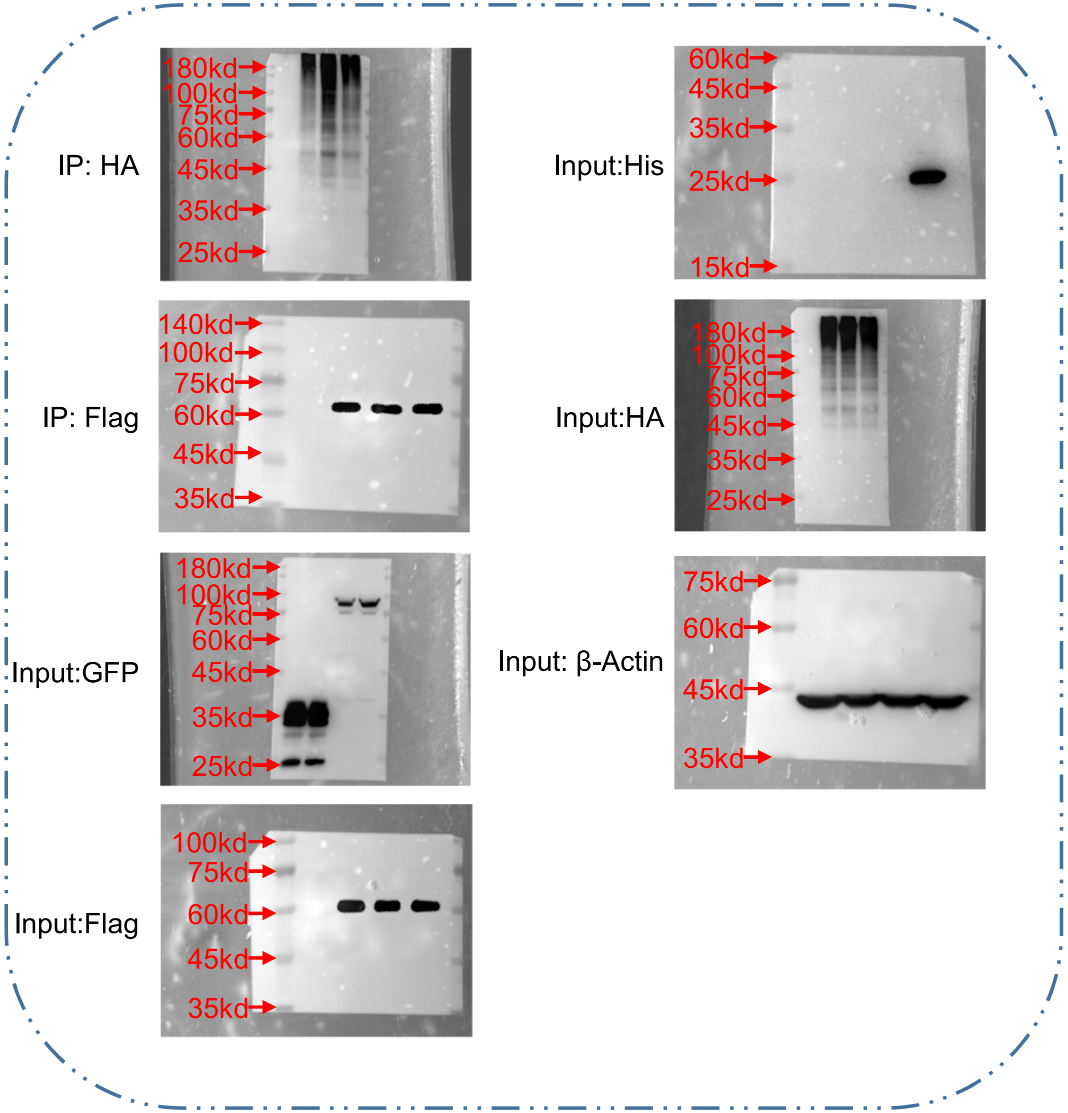

Figure S3B

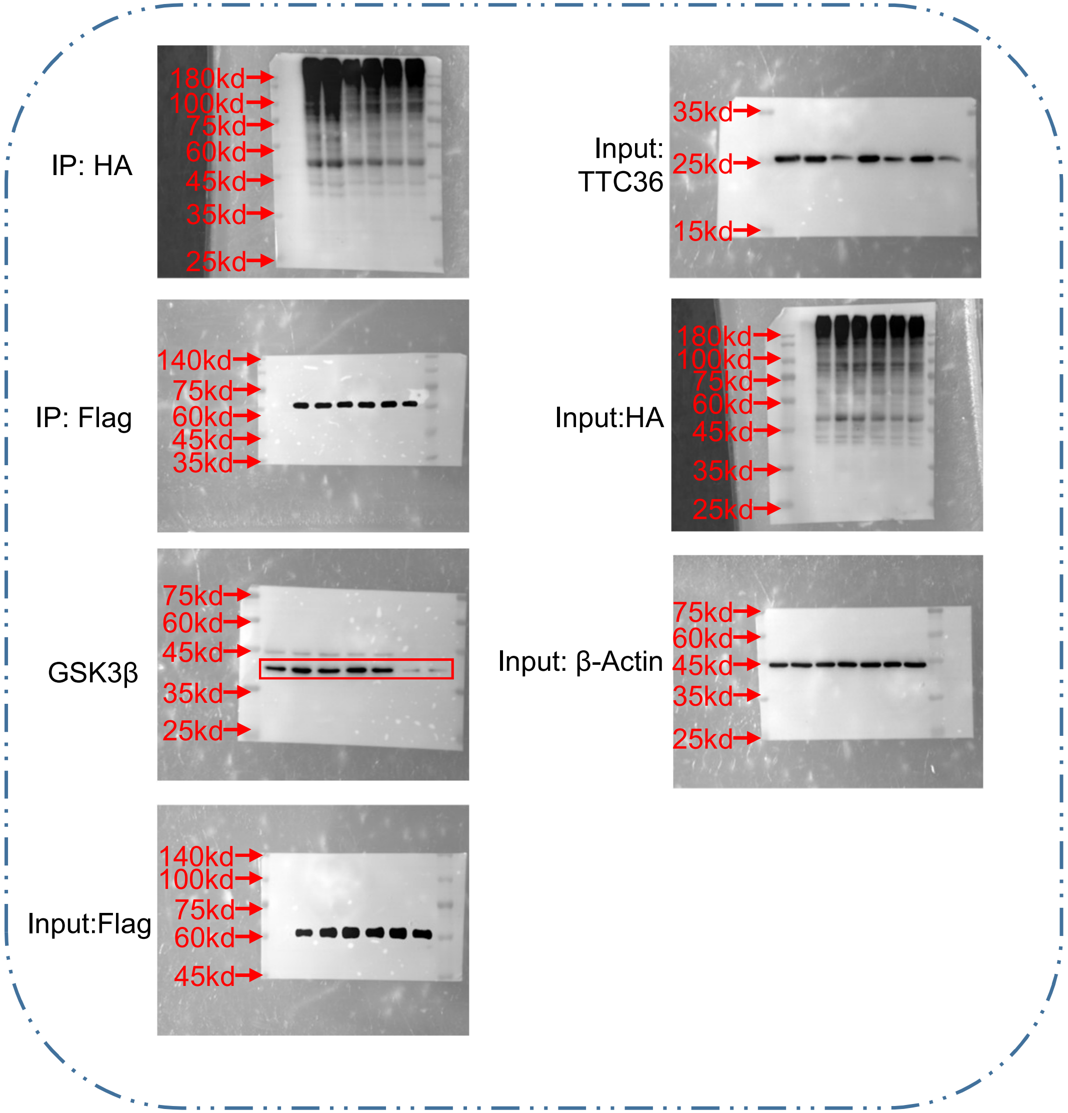

Figure S4

Figure S4A

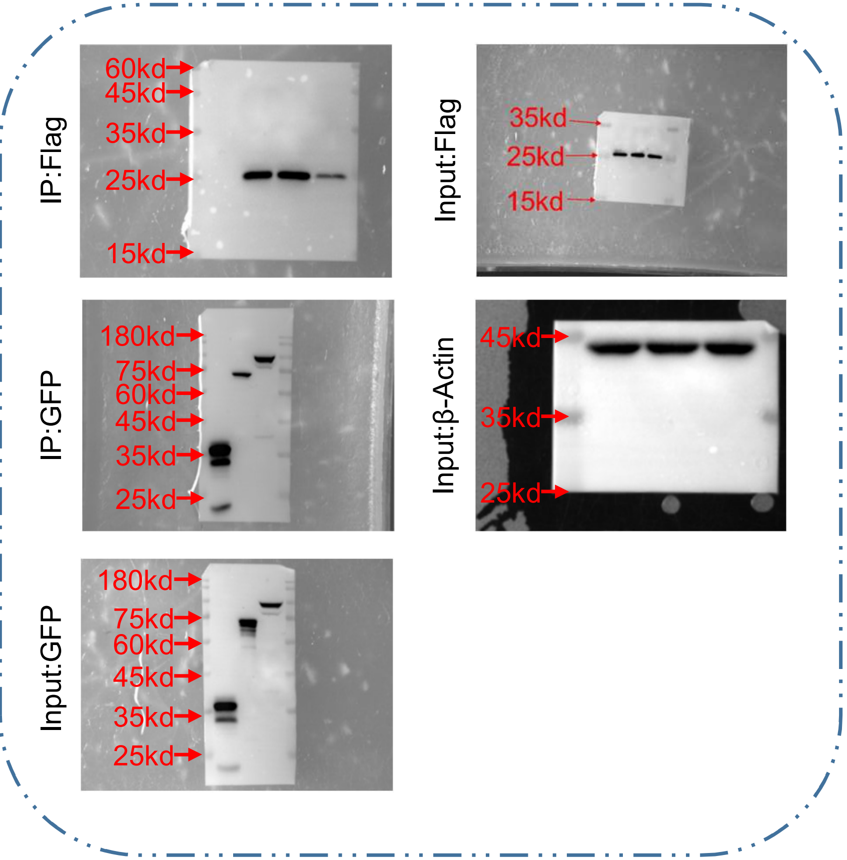

Figure S4B

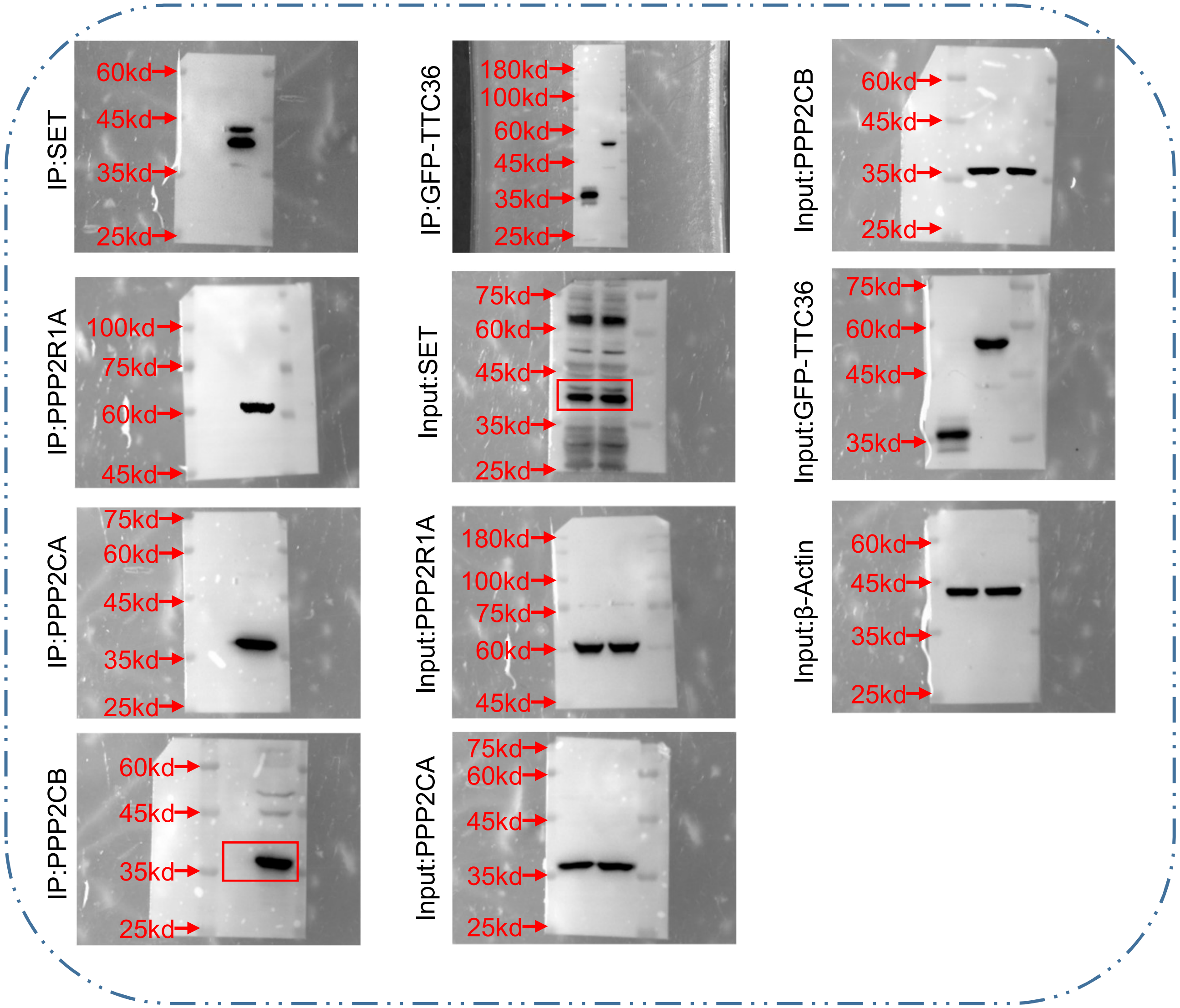

Figure S4

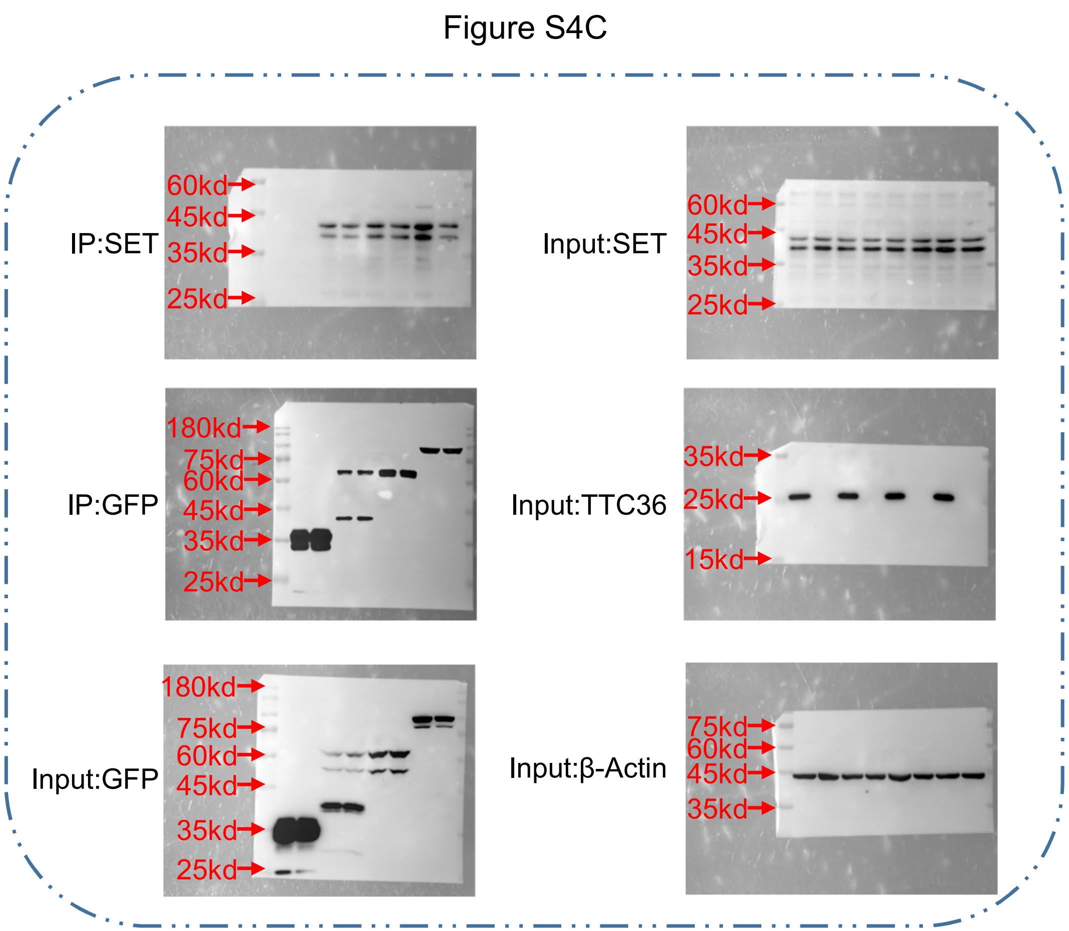
Figure S4C
IP:SET
60kd
45kd
35kd
25kd
Input:SET
60kd
45kd
35kd
25kd
IP:GFP
180kd
75kd
60kd
45kd
35kd
25kd
Input:TTC36
35kd
25kd
15kd
Input:GFP
180kd
75kd
60kd
45kd
35kd
25kd
Input:β-Actin
75kd
60kd
45kd
35kd

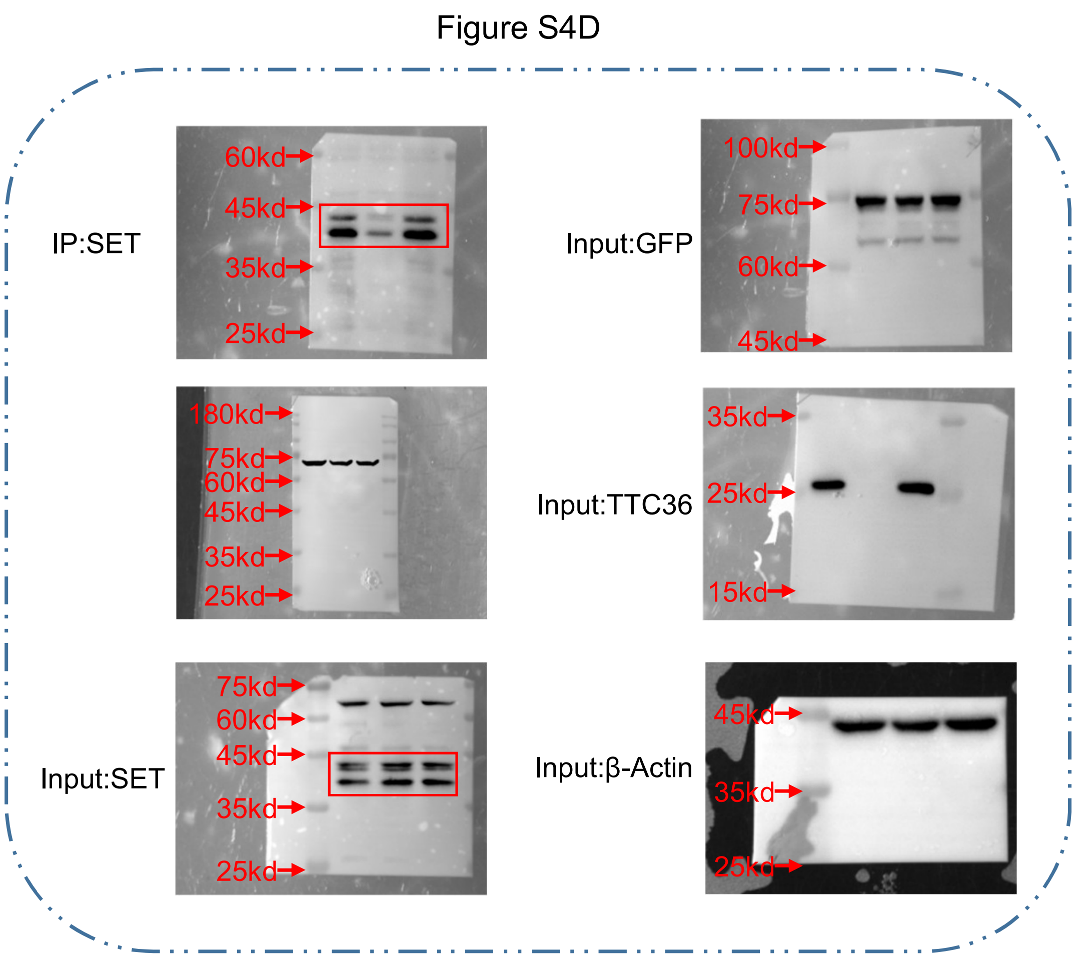
Figure S4D
IP:SET
60kd
45kd
35kd
25kd
Input:GFP
100kd
75kd
60kd
45kd
180kd
75kd
60kd
45kd
35kd
25kd
Input:TTC36
35kd
25kd
15kd
Input:SET
75kd
60kd
45kd
35kd
25kd
Input:β-Actin
45kd
35kd
25kd

Figure S4

Figure S4E

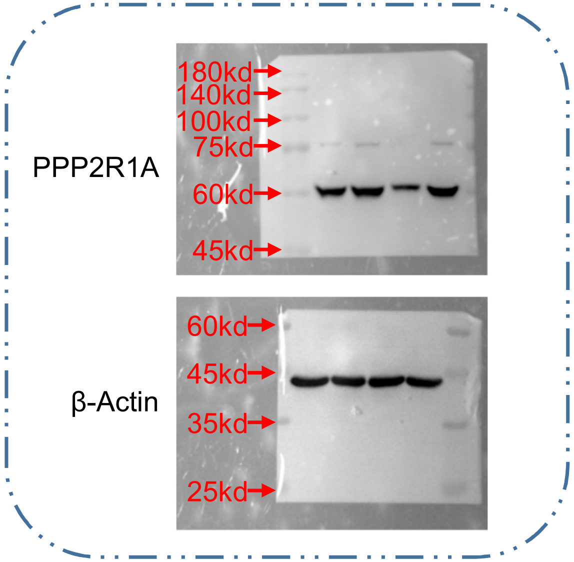

Figure S4F

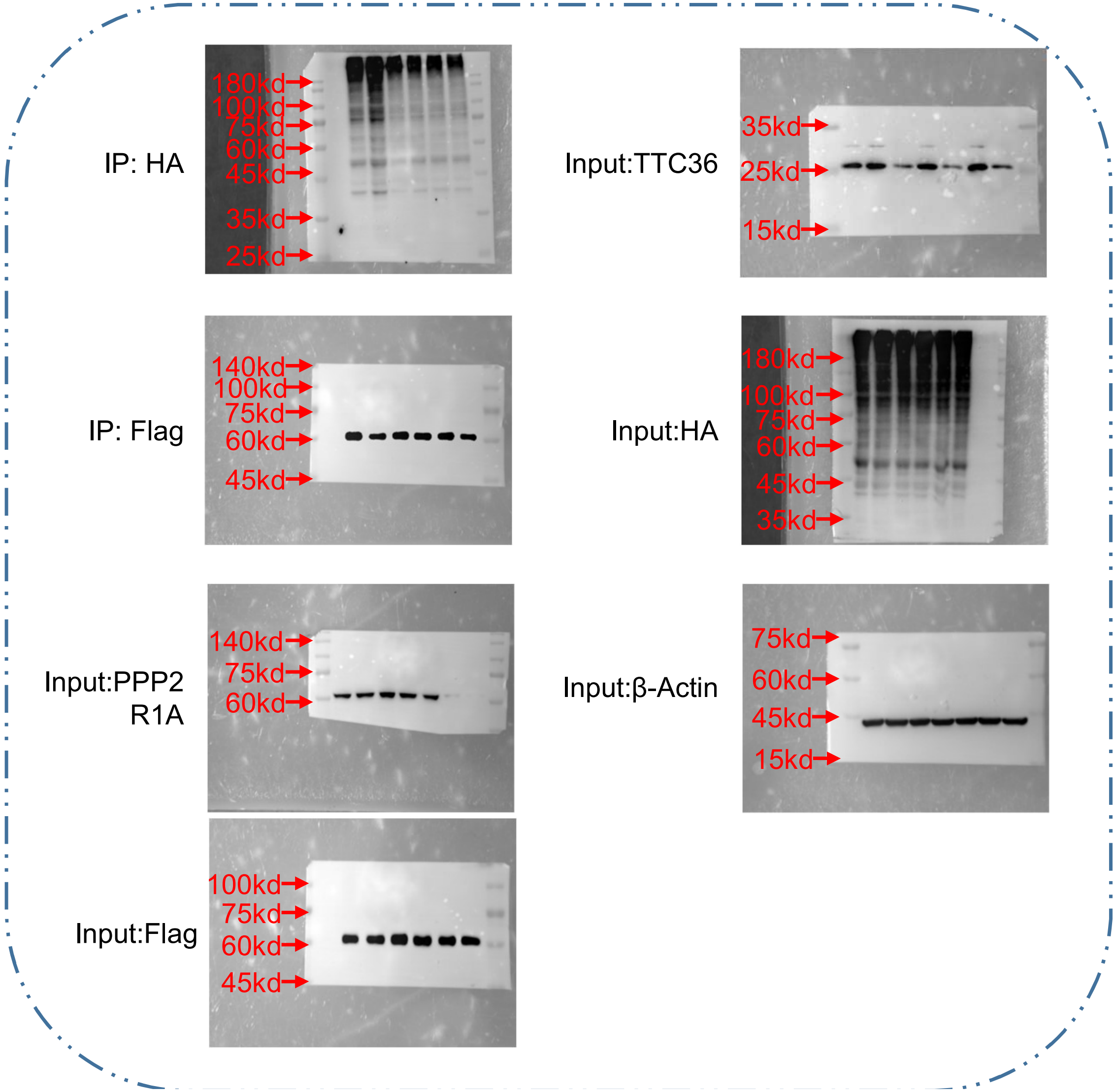

Figure S5

Figure S5A

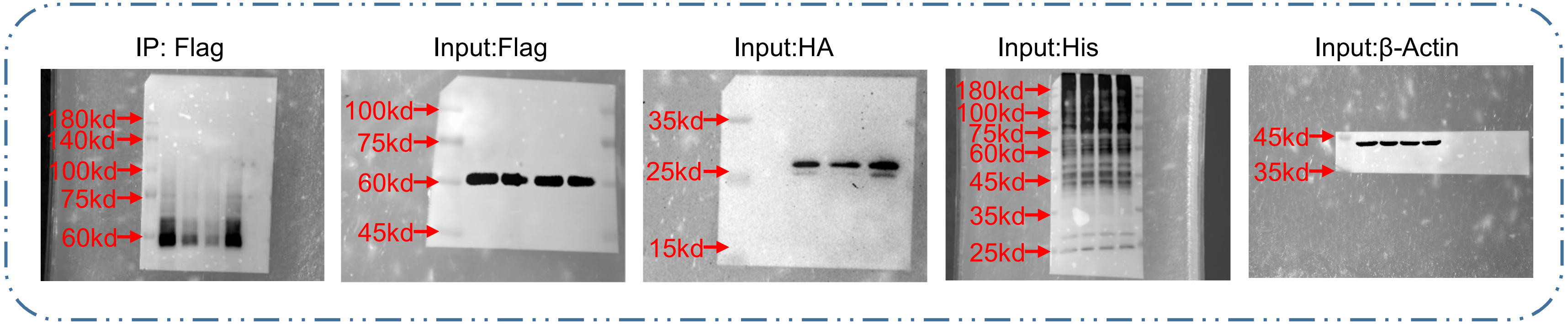

Supplement: Supplementary file 2 — Raw WB [file 41419_2025_7663_MOESM2_ESM.pdf]
